# Supplementary material for: Programmable C:G to G:C genome editing with CRISPR-Cas9-directed base excision repair proteins
Source: Nat Commun. 2021 Mar 2;12:1384. doi: 10.1038/s41467-021-21559-9 (PMC7925527; doi:10.1038/s41467-021-21559-9)
Supplement: Supplementary file 1 — Supplementary Information [file 41467_2021_21559_MOESM1_ESM.pdf]

Supplementary Information

Programmable C:G to G:C genome editing with CRISPR-Cas9-directed base excision repair proteins

Liwei Chen[1], Jung Eun Park[1], Peter Paa[1], Priscilla D. Rajakumar[1], Hong-Ting Prekop[1], Yi Ting Chew[1], Swathi N. Manivannan[1], Wei Leong Chew[1, *]

[1] Genome Institute of Singapore, Agency for Science, Technology and Research, Singapore 138672, Singapore.

*Correspondence to: chewwl@gis.a-star.edu.sg

Supplementary figures, tables and legends:

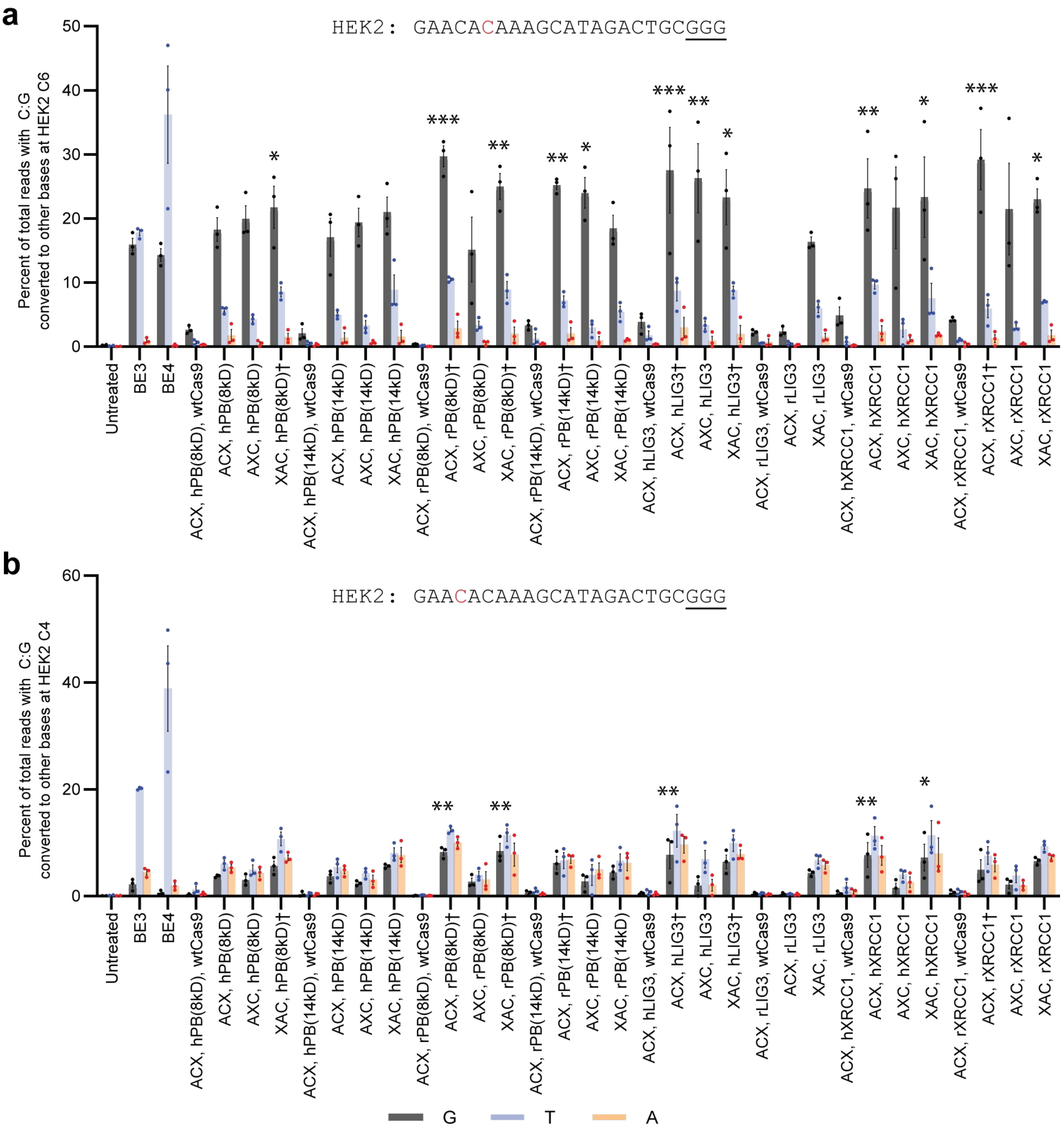

Supplementary Figure 1. Initial screen of CGBE candidates on *HEK2*. (a) For some CGBE candidates, C:G to G:C editing is the predominant edit at position 6. **(b)** C:G to T:A editing is the predominant edit at position 4. The seven candidates selected for further studies are marked with †. Targeted C's are in red. PAMs are underlined. *p < 0.05; **p < 0.01; ***p < 0.001 using one-way ANOVA (Dunn- Šidák) of C:G to G:C editing against 'Untreated.' Exact p values are available in Source Data. Each dot represents editing of an individual biological replicate; bars represent mean values; error bars represent SEM of three biologically independent replicates.

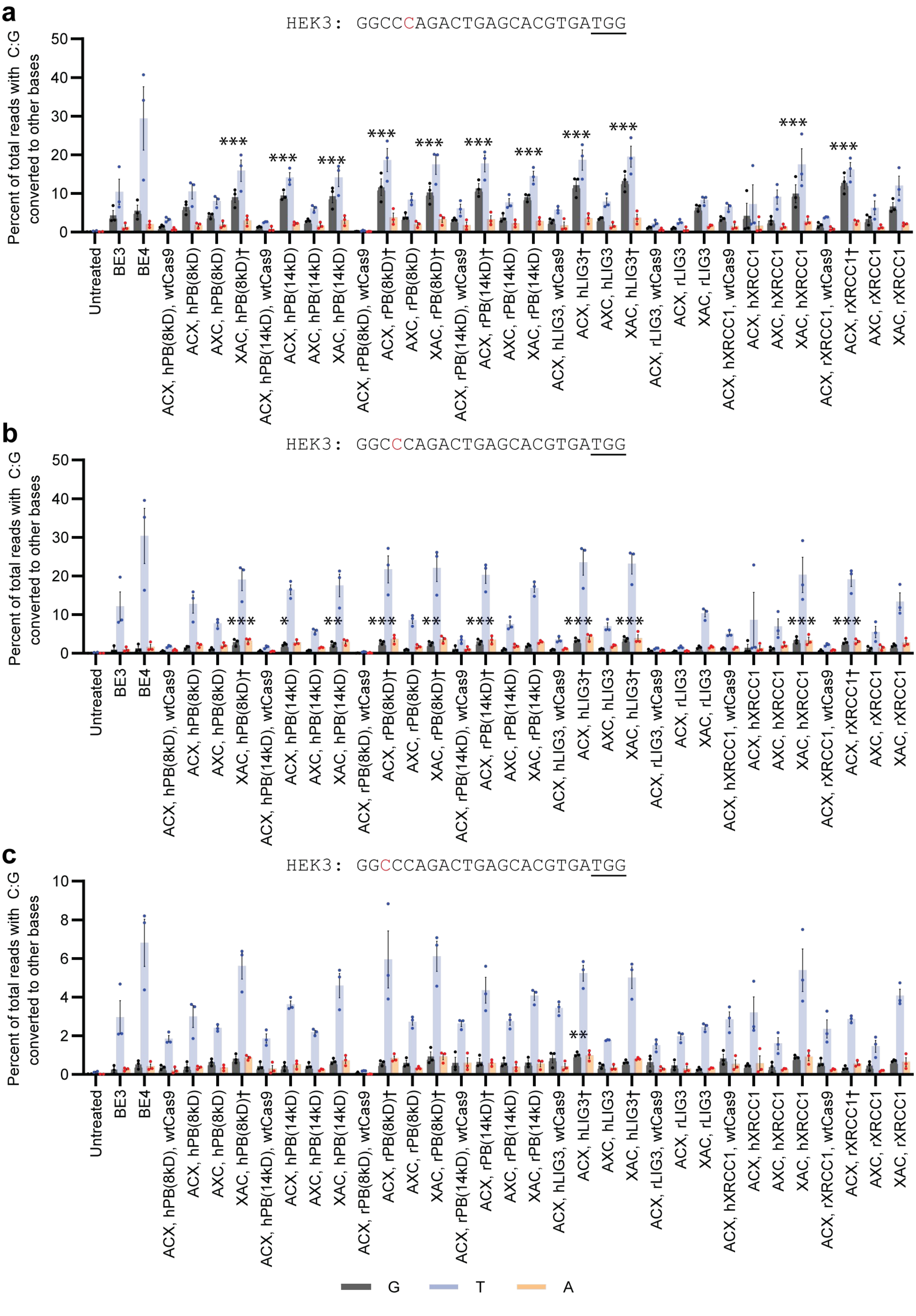

Supplementary Figure 2. Initial screen of CGBE candidates on *HEK3* at (a) position 5, (b) position 4, and (c) position 3. The seven candidates selected for further studies are marked with †. Targeted C is in red. PAM is underlined. *p < 0.05; **p < 0.01; ***p < 0.001

using one-way ANOVA (Dunn- Šidák) of C:G to G:C editing against 'Untreated.' Exact p values are available in Source Data. Each dot represents editing of an individual biological replicate; bars represent mean values; error bars represent SEM of three biologically independent replicates.

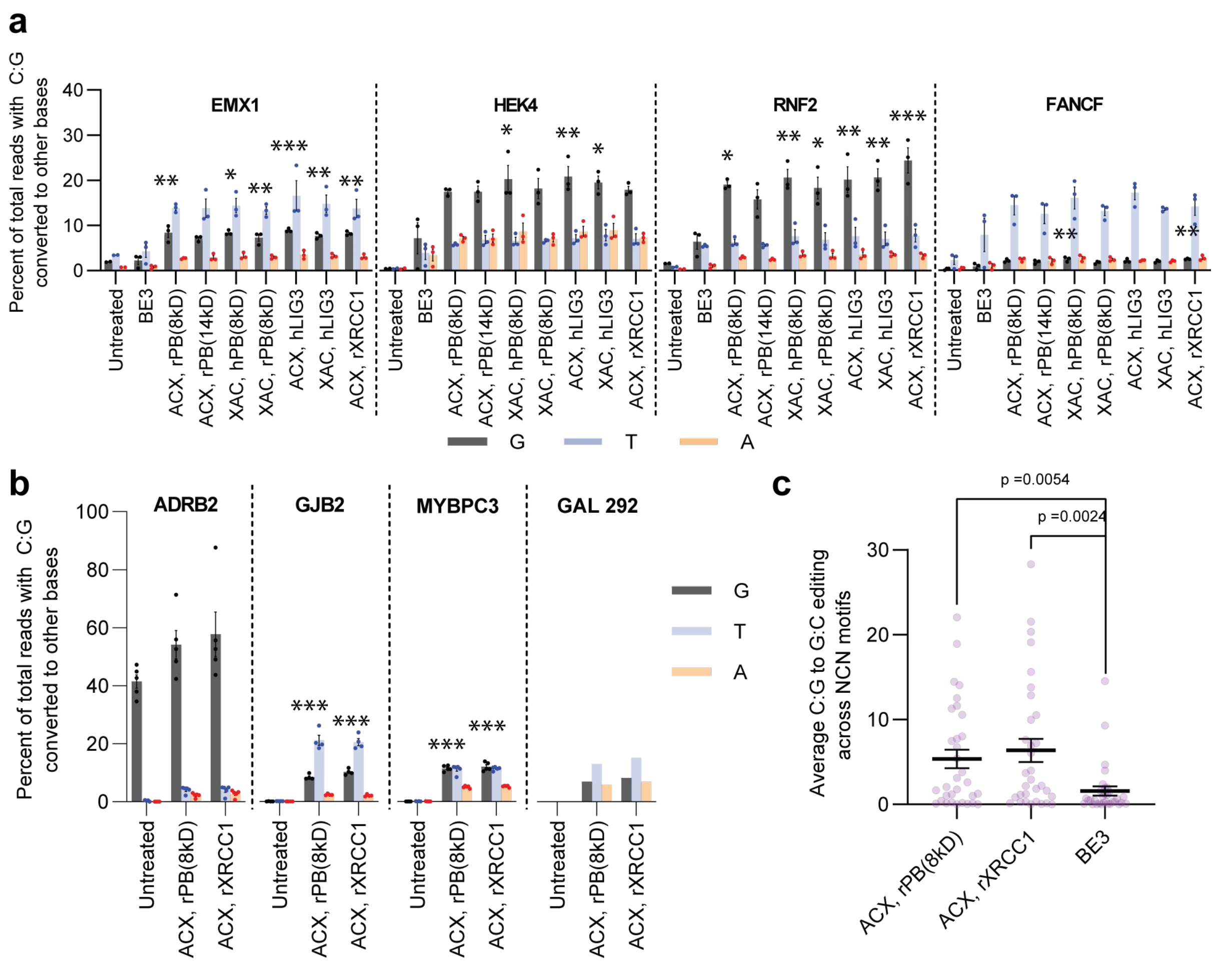

Supplementary Figure 3. Further characterization of shortlisted CGBE candidates. (a) CGBE candidates effect C:G to G:C mutations at *EMX1*, *HEK4*, *RNF2*, and *FANCF*. C:G to G:C editing is the main edit at *HEK4* and *RNF2*; C:G to T:A editing is the main edit at *FANCF* and *EMX1*. *p < 0.05; **p < 0.01; ***p < 0.001 using one-way ANOVA (Dunn-Šidák) of C:G to G:C editing against 'BE3.' Exact p values are available in Source Data. Each dot represents editing of an individual biological replicate; bars represent mean values; error bars represent SEM of three biologically independent replicates. **(b)** CGBE editing at disease-associated genes *ADRB2*, *GJB2*, *MYBPC3*, and *GAL 292*. Note that *ADRB2* contains naturally occurring polymorphism in HEK293AAV cells, and hence this data is not included in Fig 3. *p < 0.05; **p < 0.01; ***p < 0.001 using one-way ANOVA (Dunn- Šidák) of C:G to G:C editing against 'Untreated.' Exact p values are available in Source Data. Each dot represents editing of an individual biological replicate; bars represent mean values; where present, error bars represent SEM of five (*ADRB2* and *MYBPC3*) or four (*GJB2*) biologically independent replicates. **(c)** Mean C:G to G:C editing as a percent of all reads across 16 NCN sites. CGBEs increase C:G to G:C editing by three to four fold compared to BE3, across all possible NCN sequences. p values were obtained via Mann-Whitney tests between indicated editors. Each dot represents C:G to G:C editing with one of the 16 *HEK2* gRNAs in an individual biological replicate. Sequences for the 16 gRNAs are listed in Supplementary Table 2. Lines represent mean values and SEM of 32 biologically independent replicates – each of the 16 gRNAs is tested twice.

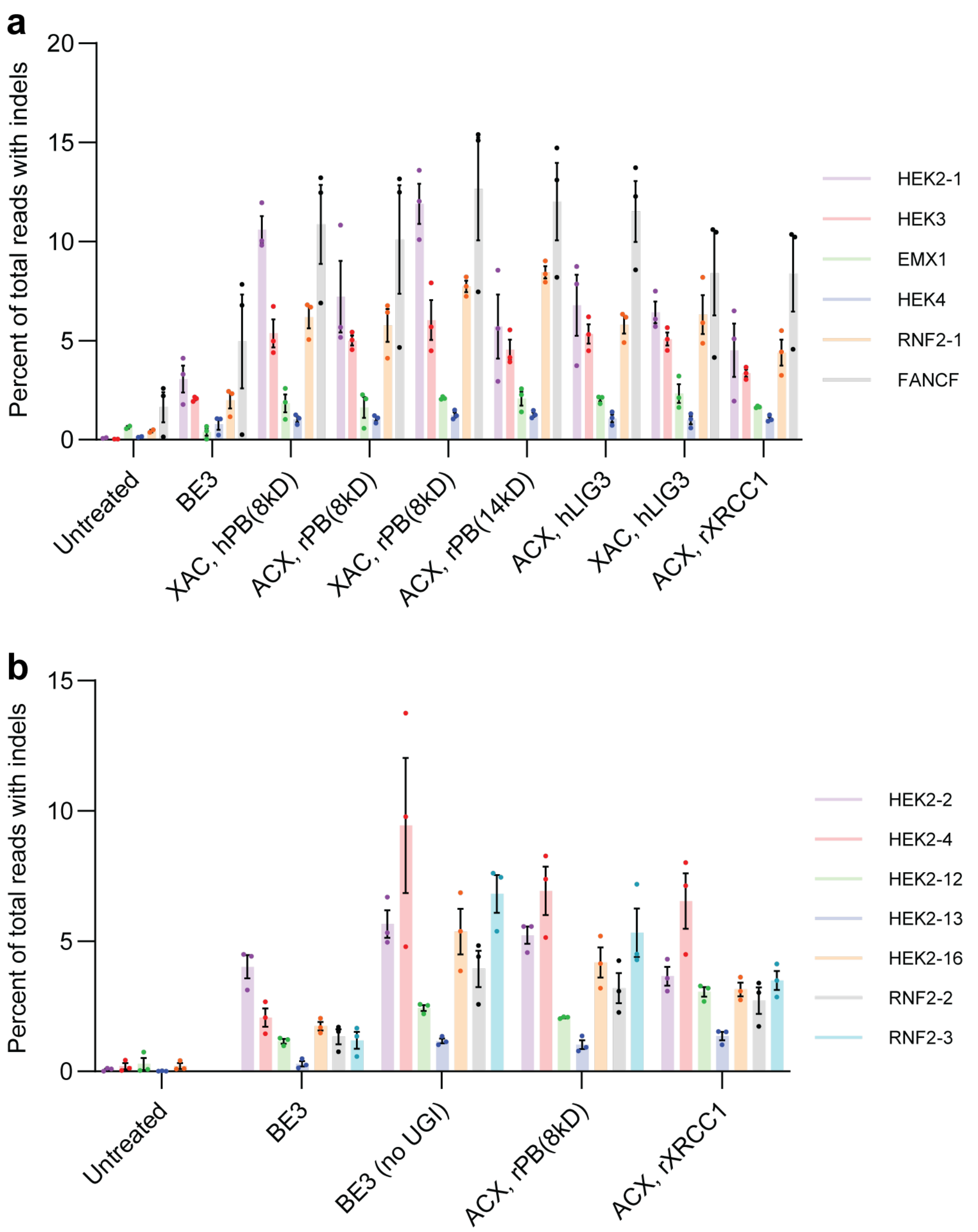

Supplementary Figure 4. Indel rates of shortlisted CGBE candidates at genomic sites. **(a)** CGBE candidates generate higher indel rates than BE3. ACX, rXRCC1 has the lowest indel rate of CGBE candidates. **(b)** Removing UGI from BE3 increases indel rate; fusing BER proteins rPB(8kD) or rXRCC1 decreases indel rate modestly. An additional set of gRNA:targets used here validates the conclusion from (a).While further mechanistic studies would be necessary, a possible hypothesis is that recruitment of the BER complex repairs abasic sites and the shortened persistence of these abasic sites may then lead to a lower propensity for indels. For both plots, each dot represents indels of an individual biological replicate; bars represent mean values; error bars represent SEM of three biologically independent replicates.

a ADRB2

WT	C_1	C_2	C_3	T_4	T_5	T_6	C_7	C_8	T_9	G_{10}	C_{11}	G_{12}	T_{13}	G_{14}	A_{15}	C_{16}	G_{17}	T_{18}	C_{19}	G_{20}
A	0.0	0.0	0.0	0.1	0.0	0.0	0.1	0.0	0.0	0.1	0.0	0.2	0.1	0.1	99.8	0.0	0.3	0.0	0.0	0.2
C	99.9	99.9	99.8	0.2	0.2	0.1	54.5	99.4	0.1	0.1	99.8	0.4	0.1	0.1	0.0	99.9	0.1	0.4	99.9	0.1
G	0.0	0.0	0.0	0.1	0.1	0.0	44.9	0.0	0.1	99.7	0.0	99.5	0.2	99.7	0.2	0.0	99.6	0.3	0.0	99.5
T	0.1	0.1	0.1	99.6	99.7	99.9	0.6	0.5	99.7	0.1	0.2	0.0	99.5	0.0	0.1	0.1	0.1	99.3	0.1	0.2

XRCC	C_1	C_2	C_3	T_4	T_5	T_6	C_7	C_8	T_9	G_{10}	C_{11}	G_{12}	T_{13}	G_{14}	A_{15}	C_{16}	G_{17}	T_{18}	C_{19}	G_{20}
A	0.1	0.0	0.1	0.0	0.1	0.0	0.7	0.1	0.0	0.1	0.0	0.3	0.0	0.1	99.8	0.0	0.2	0.0	0.0	0.3
C	99.6	99.9	99.6	0.2	0.1	0.1	10.6	99.2	0.1	0.1	99.8	0.2	0.1	0.1	0.0	99.9	0.0	0.3	99.9	0.1
G	0.0	0.0	0.0	0.2	0.1	0.1	87.6	0.1	0.1	99.8	0.0	99.4	0.3	99.8	0.1	0.0	99.6	0.2	0.0	99.5
T	0.3	0.1	0.3	99.6	99.7	99.8	1.1	0.6	99.8	0.1	0.2	0.0	99.6	0.0	0.1	0.1	0.1	99.4	0.1	0.2

rPB	C_1	C_2	C_3	T_4	T_5	T_6	C_7	C_8	T_9	G_{10}	C_{11}	G_{12}	T_{13}	G_{14}	A_{15}	C_{16}	G_{17}	T_{18}	C_{19}	G_{20}
A	0.1	0.0	0.1	0.0	0.1	0.0	1.0	0.3	0.1	0.1	0.0	0.2	0.1	0.1	99.7	0.0	0.3	0.0	0.0	0.2
C	99.5	99.7	99.1	0.2	0.1	0.1	25.4	98.5	0.1	0.1	99.8	0.3	0.1	0.1	0.0	99.9	0.1	0.4	99.9	0.1
G	0.0	0.0	0.0	0.1	0.1	0.1	71.4	0.1	0.1	99.8	0.0	99.5	0.2	99.8	0.2	0.0	99.5	0.3	0.0	99.6
T	0.4	0.3	0.7	99.6	99.7	99.8	2.2	1.1	99.7	0.1	0.2	0.0	99.6	0.1	0.1	0.1	0.1	99.3	0.1	0.1

b MYBPC3

WT	G_1	A_2	T_3	A_4	G_5	A_6	C_7	C_8	T_9	G_{10}	T_{11}	G_{12}	T_{13}	G_{14}	C_{15}	A_{16}	T_{17}	G_{18}	G_{19}	A_{20}
A	0.1	99.8	0.7	99.8	0.1	99.9	0.1	0.0	2.3	1.0	0.7	7.2	0.1	0.1	0.0	99.9	1.4	3.0	0.2	99.9
C	0.0	0.0	0.0	0.0	0.1	0.0	99.7	99.6	0.1	0.0	0.0	0.0	0.0	0.0	99.2	0.0	0.0	0.0	0.0	0.0
G	99.8	0.1	0.0	0.1	99.8	0.1	0.1	0.3	0.0	99.0	0.0	92.8	0.0	99.9	0.4	0.0	0.0	97.0	99.8	0.1
T	0.0	0.0	99.3	0.1	0.0	0.0	0.1	0.1	97.6	0.0	99.3	0.0	99.8	0.0	0.3	0.1	98.5	0.0	0.0	0.0

XRCC	G_1	A_2	T_3	A_4	G_5	A_6	C_7	C_8	T_9	G_{10}	T_{11}	G_{12}	T_{13}	G_{14}	C_{15}	A_{16}	T_{17}	G_{18}	G_{19}	A_{20}
A	0.2	99.7	0.9	99.8	0.2	99.8	5.3	2.0	2.1	1.1	0.6	7.2	0.1	0.1	0.0	99.9	1.1	3.0	0.2	99.9
C	0.2	0.1	0.1	0.0	0.1	0.0	69.4	94.0	0.1	0.1	0.0	0.0	0.1	0.0	99.3	0.0	0.0	0.0	0.0	0.0
G	99.6	0.1	0.0	0.1	99.7	0.1	13.9	1.3	0.0	98.8	0.0	92.7	0.0	99.9	0.4	0.0	0.0	97.0	99.8	0.1
T	0.0	0.0	99.0	0.1	0.0	0.1	11.3	2.7	97.8	0.0	99.3	0.1	99.8	0.0	0.3	0.1	98.8	0.0	0.0	0.0

rPB	G_1	A_2	T_3	A_4	G_5	A_6	C_7	C_8	T_9	G_{10}	T_{11}	G_{12}	T_{13}	G_{14}	C_{15}	A_{16}	T_{17}	G_{18}	G_{19}	A_{20}
A	0.3	99.7	0.9	99.7	0.2	99.8	5.2	2.4	2.2	1.2	0.6	7.6	0.1	0.1	0.1	99.9	1.2	2.9	0.2	99.9
C	0.1	0.1	0.1	0.0	0.1	0.0	71.1	92.9	0.1	0.1	0.1	0.0	0.0	0.0	99.2	0.0	0.0	0.0	0.0	0.0
G	99.6	0.1	0.0	0.1	99.7	0.1	12.3	1.4	0.0	98.7	0.0	92.3	0.1	99.9	0.4	0.0	0.0	97.1	99.8	0.1
T	0.0	0.1	99.0	0.2	0.0	0.1	11.3	3.3	97.7	0.0	99.3	0.1	99.8	0.0	0.3	0.1	98.8	0.0	0.0	0.0

Supplementary Figure 5. Representative data of CGBE editing at (a) *ADRB2* and (b) *MYBPC3*. WT denotes wild-type untreated cells; XRCC denotes ACX, XRCC1; rPB denotes ACX, rPB(8kD). Note that *ADRB2* contains naturally occurring polymorphism in HEK293AAV cells.

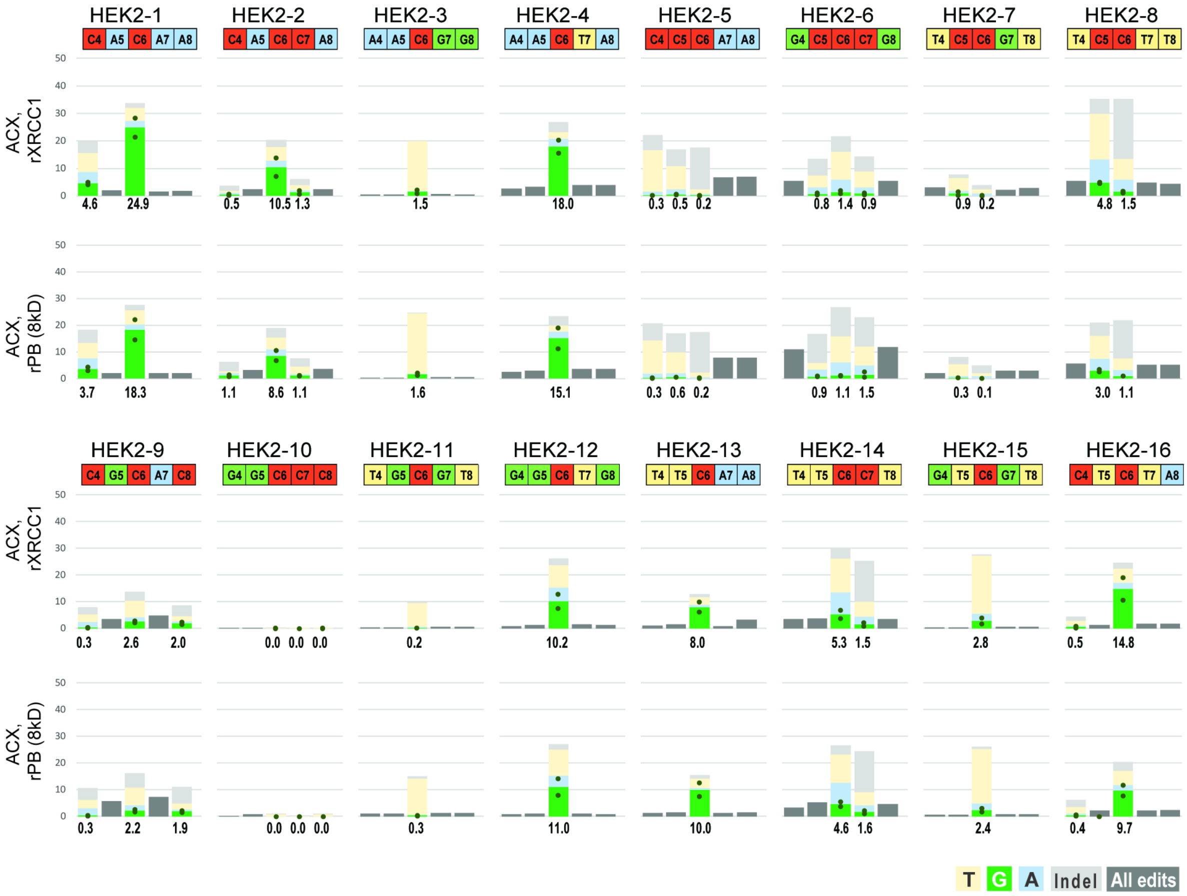

Supplementary Figure 6. C:G to G:C editing for ACX, rXRCC1 and ACX, rPB(8kD) across 16 NCN gRNA:target combinations. Editing frequencies on all nucleotides within positions 4 to 8 are depicted, with C6 data reported in Fig 2a. When targeting C's at position 6 (24.9%), the highest bystander C:G to G:C edit observed is 4.8% (HEK2-1, position 4). Full sequences of target sites are available in Supplementary Table 2. Each dot represents C:G to G:C editing of an individual biological replicate; bars represent mean values of two biologically independent replicates.

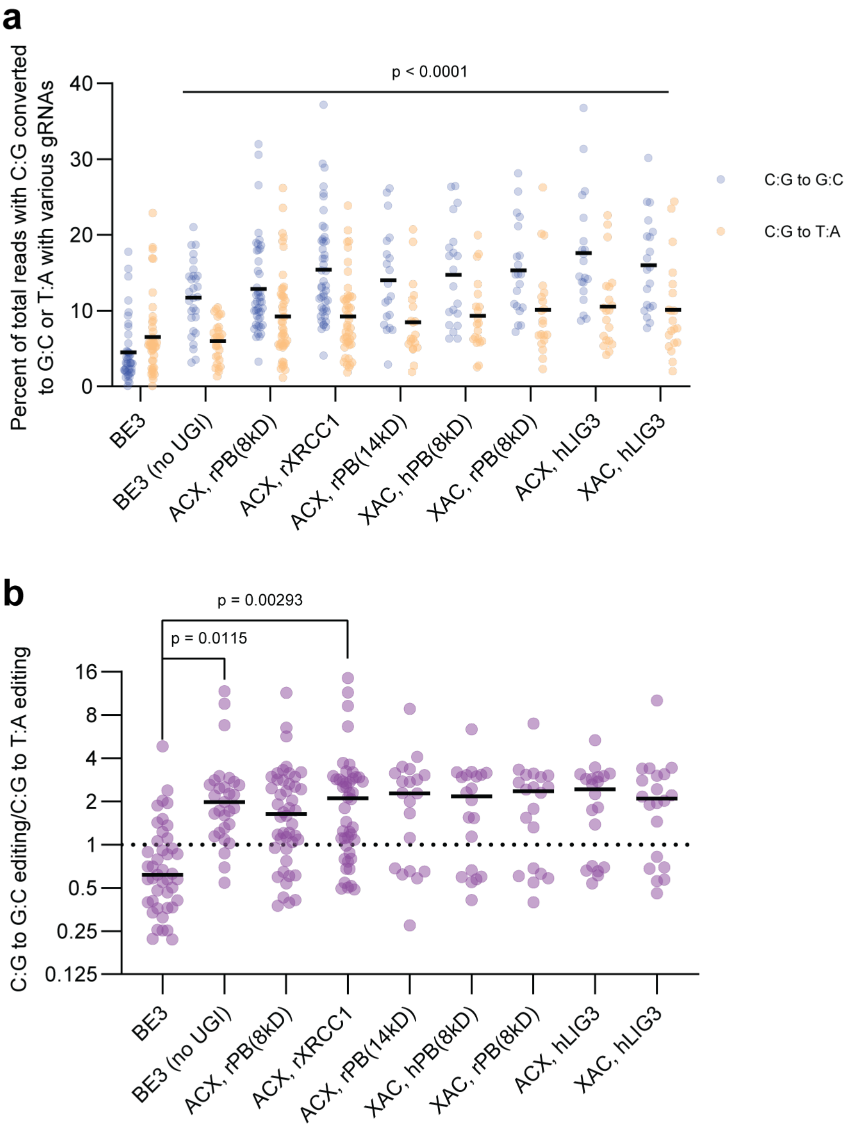

Supplementary Figure 7. ACX, rXRCC1 is the best performer out of shortlisted CGBE candidates. (a) C:G to G:C editing (blue) vs. C:G to T:A editing (orange) as percent of all reads across gRNAs used in this study. All biological replicates are included except those targeting the 10 suboptimal C:G to G:C base editing motifs (Figure 2a and Figure 2b) and *ADRB2* due to naturally occurring polymorphism (Supplementary Figure 3b). **(b)** Ratio of C:G to G:C editing to C:G to T:A editing across gRNAs used in this study. Only BE3 (no UGI) and ACX, rXRCC1 give a significantly higher ratio of C:G to G:C editing/C:G to T:A editing. For both plots, p values were obtained via one-way ANOVA (Dunn-Šidák) against 'BE3.' Each dot represents editing of an individual biological replicate. Black lines represent mean values of 43 (BE3), 29 (BE3 (no UGI)), 46 (ACX, rPB(8KD) and ACX, rXRCC1), or 20 (the remaining editors) biologically independent replicates.

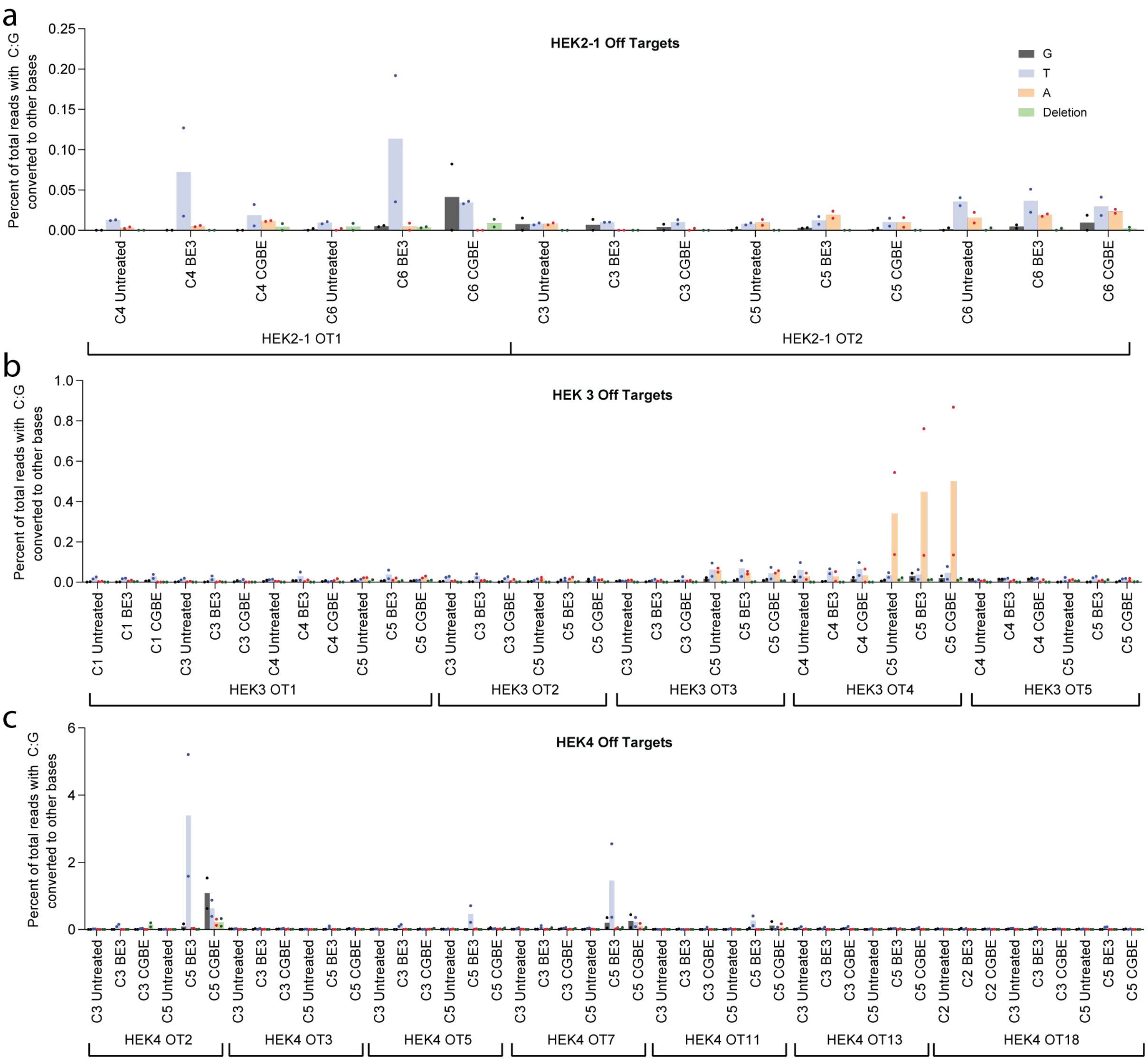

Supplementary Figure 8. Continued on next page

d

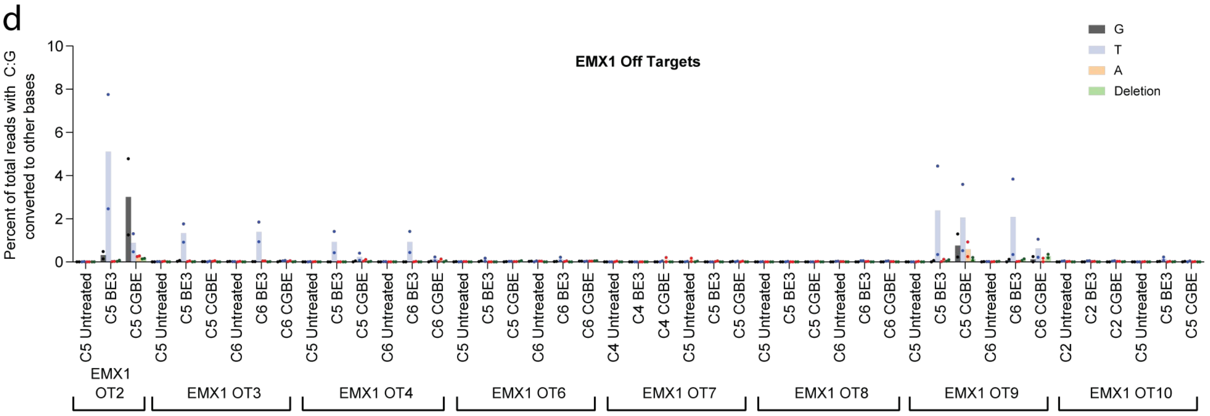

e

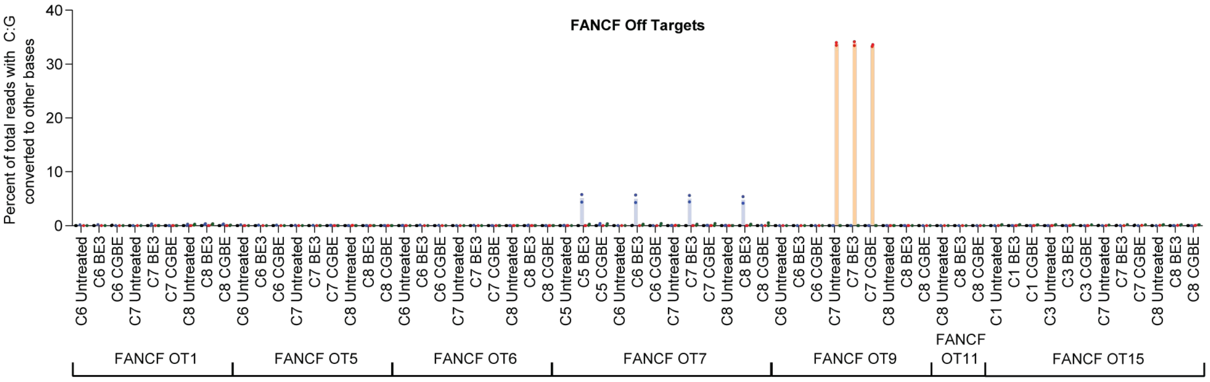

Supplementary Figure 8. CGBE and BE3 off target activity at identified off-target sites with (a) *HEK2-1* gRNA; (b) *HEK3* gRNA; (c) *HEK4* gRNA; (d) *EMX1*; and (e) *FANCF*. A total of 29 identified off-target sites with 68 editable C's using 5 gRNAs were tested. *HEK3* and *EMX1* off-target sites are Cas9 off-target sites identified via GUIDE-Seq[1]; *HEK2*, *HEK4*, and *FANCF* off-target sites are BE3 (no UGI) off-target sites identified via Digenome-Seq[2]. CGBE and BE3 induced >0.1% C:G to D:H edits at the same 15 off-target sites. At 2 out of these 15 positions, CGBE induced greater off-target editing frequency than BE3; at the remaining 13 sites, CGBE induced lower off-target editing frequency. 'OT5' indicates off-target 5; 'C4' indicates a 'C' at position 4. Each dot represents editing of an individual biological replicate; bars represent mean values of two biologically independent replicates.

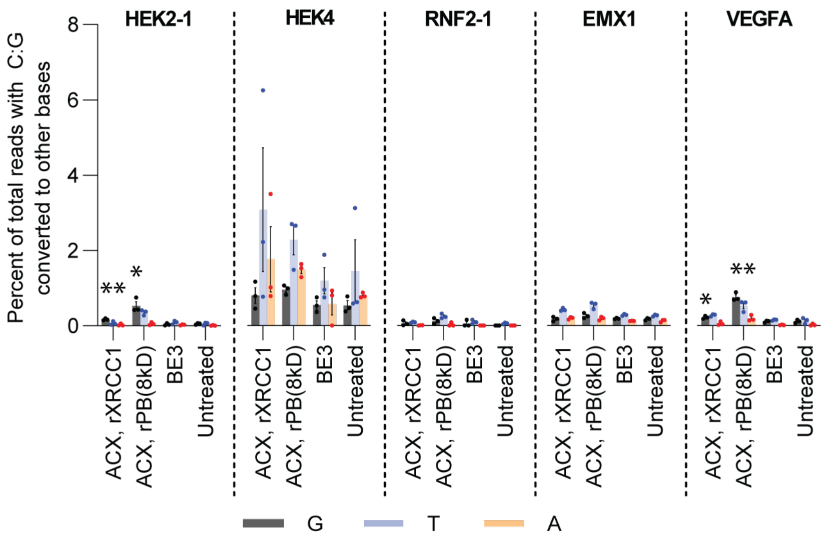

Supplementary Figure 9. ACX, rXRCC1; ACX, rPB(8kD); and BE3 exhibit low editing efficiencies in H9 stem cells. Without further engineering (via codon optimization, APOBEC mutation etc.), BE3 is inefficient at inducing C:G to T:A edits in H9 stem cells. The highest C:G to T:A editing observed with BE3 is at HEK4 (1.2%). Similarly, both CGBEs are not efficient at inducing C:G to G:C edits, with the highest edits also at *HEK4*. Zeng et al recently showed that the engineered human APOBEC3A can increase BE3 editing in stem cells[3], suggesting that a similar approach might also induce higher CGBE stem cell editing. *p < 0.05; **p < 0.01 using two-tailed Student's t Test of C:G to G:C editing against 'Untreated.' Exact p values are available in Source Data. Each dot represents editing of an individual biological replicate; bars represent mean values; error bars represent SEM of three biologically independent replicates.

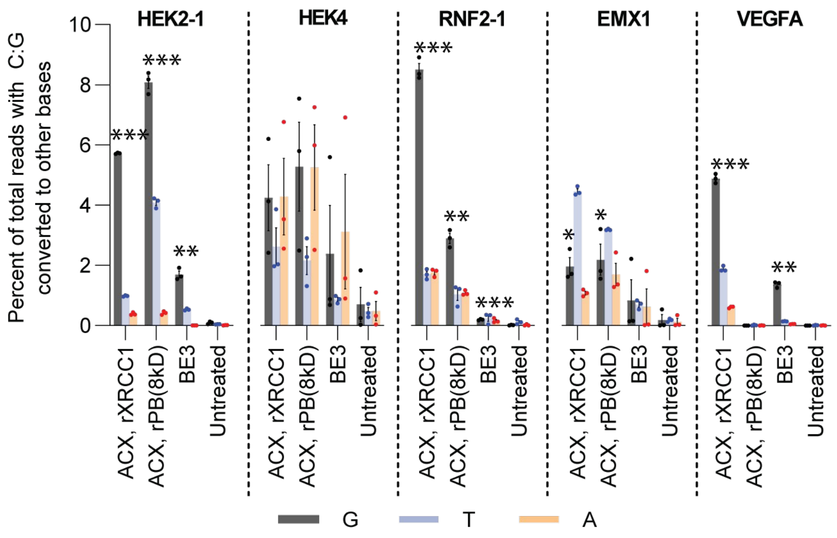

Supplementary Figure 10. ACX, rXRCC1; ACX, rPB(8kD); and BE3 editing in eHAP cells. Although BE3 editing is low, we observed moderate levels of editing with both CGBEs. These results suggest that CGBE may be able to induce some C:G to G:C edits in certain circumstances under which different base editing technology – like BE3 – may not be as efficient (C:G to T:A edits; light blue). *p < 0.05; **p < 0.01; ***p < 0.001 using two-tailed Student's t Test of C:G to G:C editing against 'Untreated.' Exact p values are available in Source Data. Each dot represents editing of an individual biological replicate; bars represent mean values; error bars represent SEM of three biologically independent replicates.

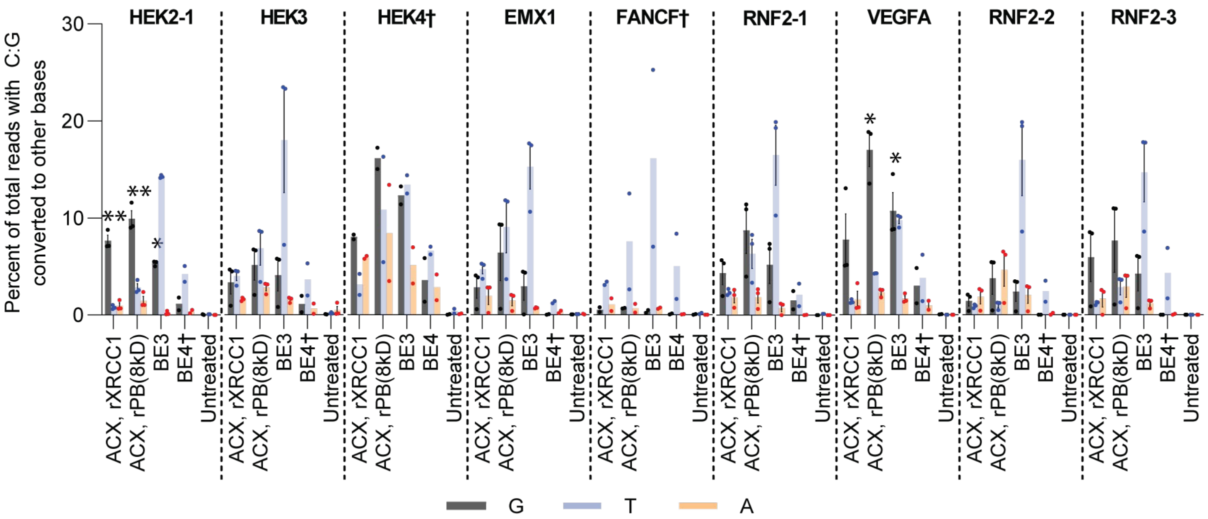

Supplementary Figure 11. ACX, rXRCC1; ACX, rPB(8kD); and BE3 editing in HTB9 cells. CGBEs are able to efficiently induce C:G to G:C edits at *HEK2-1*, *HEK4*, *RNF2-1*, and VEGFA. Additionally, C6 editing (*RNF2-3*) appears to be higher than C5 editing (*RNF2-2*). Collectively, the data indicate that the editing preferences of CGBEs in HEK cells carry over to HTB9 cells. The more efficient CGBE in HTB9 cells is ACX, rPB(8kD). *$p < 0.05$; **$p < 0.01$; ***$p < 0.001$ using two-tailed Student's t Test of C:G to G:C editing against 'Untreated.' Exact p values are available in Source Data. Each dot represents editing of an individual biological replicate; bars represent mean values; where present, error bars represent SEM of three biologically independent replicates († indicates two biologically independent repeats).

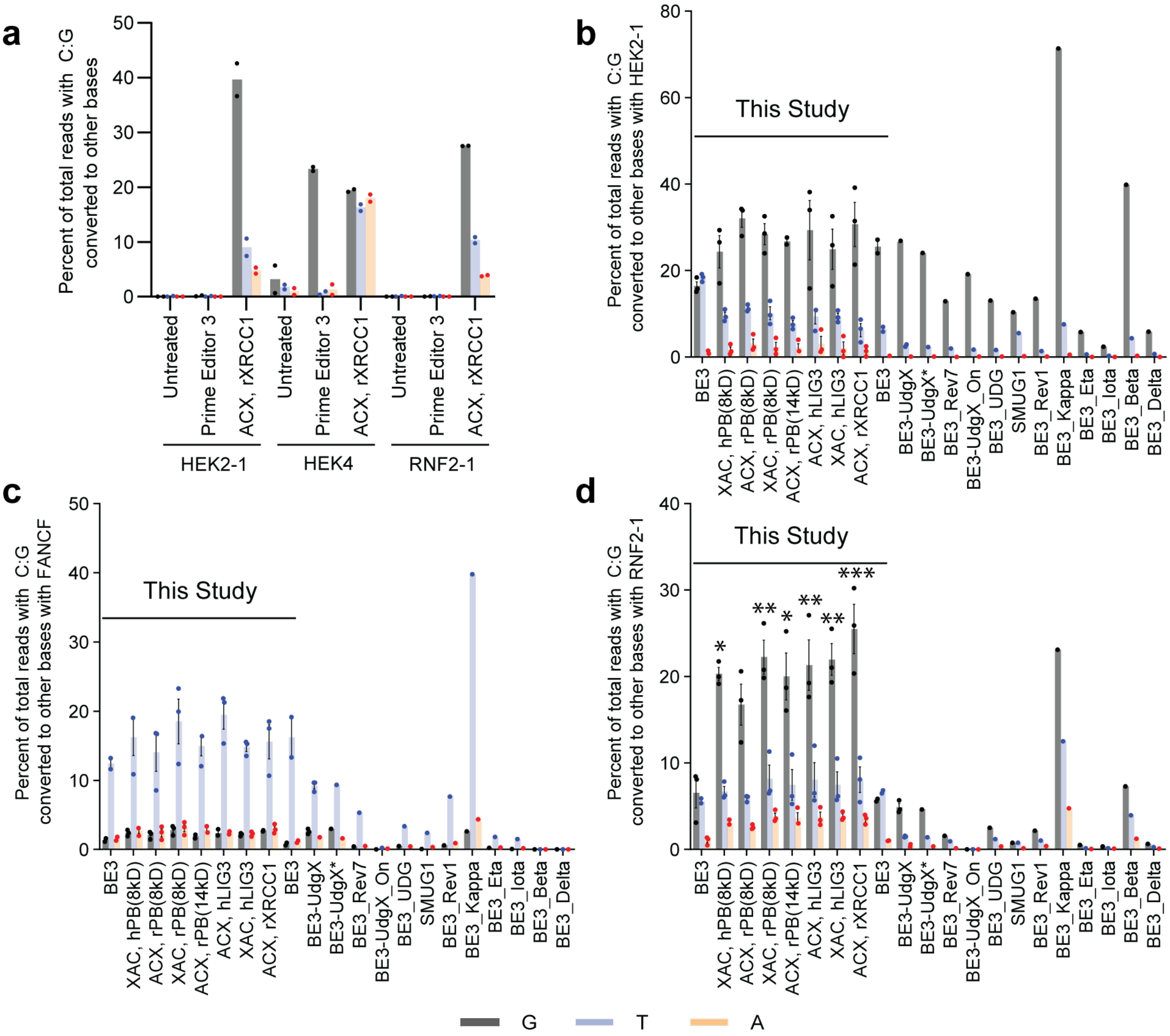

Supplementary Figure 12. Comparison of our CGBE to (a) PE3 described in Anzalone et al., and CGBEs described in Liu and Koblan at (b) *HEK2*, (c) *FANCF*, and (d) *RNF2*. (a) For C:G to G:C editing, PE3 is as efficient as CGBE (ACX, rXRCC1) and induced lower levels of undesired edits at *HEK4*. At *HEK2-1* and *RNF2-1*, PE3 is substantially less efficient than CGBE at *HEK2* and *RNF2*. Our results indicate that while PE3 may be able to perform C:G to G:C transversions at some sites, CGBE is a valuable tool to expand the editing capabilities of current technologies For parts b, c, and d, since datasets were generated independently and in different cell types, comparisons should be made only against BE3 common to the two studies. Fusion of base excision enzymes such as UDG and UdgX decreases C:G to G:C editing beyond BE3 at 2 of 3 sites (n=1; Liu and Koblan[4]). Fusion of base excision repair enzymes such as rXRCC1 increases C:G to G:C editing beyond BE3. For part d, *p < 0.05; **p < 0.01; ***p < 0.001 one-way ANOVA (Dunn-Šidák) of C:G to G:C editing against 'BE3.' For all plots, each dot represents editing of an individual biological replicate; bars represent mean values; where present, error bars represent SEM of three biologically independent samples.

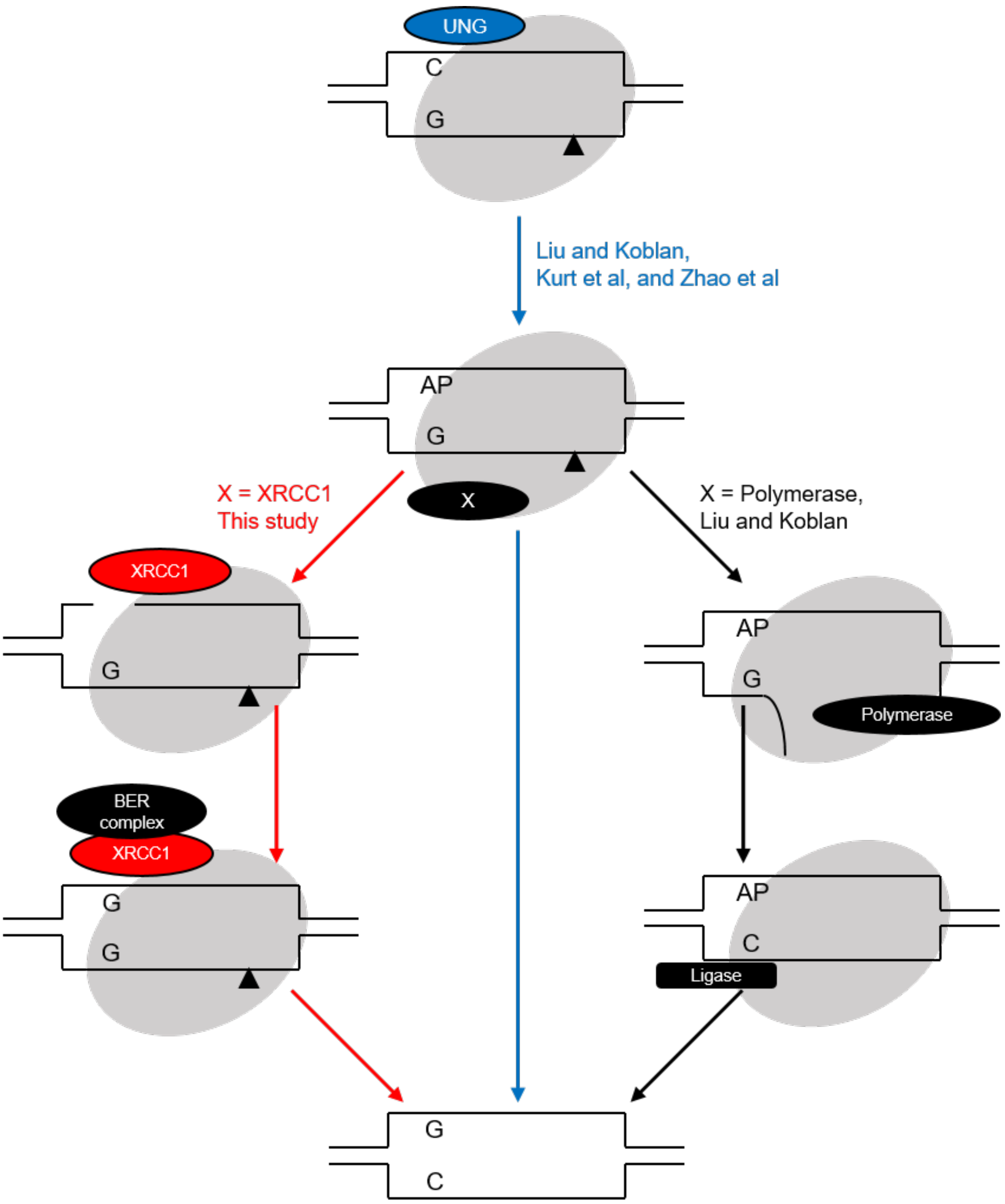

Supplementary Figure 13. Distinct strategies for CGBE design. This study employs a CGBE design strategy (left, red arrows) where Cas9 is fused to protein(s) involved in repairing uracil-containing or abasic sites (AP). The activities of these BER proteins are expected to convert the AP to G before the nucleotide on the opposite strand is converted from G to C. In contrast, the polymerase strategy employed by Liu and Koblan[4] (right, black arrows) seeks to maintain the abasic site throughout a translesion synthesis envisioned to occur on the opposite strand. The AP is repaired after the nucleotide on the opposite strand is converted from G to C. The UNG-based CGBE strategy employed by Kurt et al[5], Zhao et al[6], and Liu and Koblan seeks to facilitate the generation of the AP site (middle, blue arrows). In other words, this study employs proteins that repair and not maintain/generate abasic sites whereas other studies employ proteins that generate/maintain and not repair abasic sites. CGBEs were designed based on working hypotheses derived from known Cas9 and BER chemistries that warrants future mechanistic studies.

Supplementary Table 1. CGBEs enable potential treatment avenues to previously unaddressable SNPs associated with human diseases. CBE enables treatment to 48% of all known disease-associated SNPs in ClinVar, while ABE enables treatment to 6%. CGBEs effect primarily C:G to G:C and G:C to C:G changes (yellow highlight) that can correct 11% of disease-associated SNPs. In combination with CBEs or ABEs, CGBEs effect secondarily G to T, C to A, A to C, and T to G edits (green highlight). With CBEs, ABEs, and CGBEs, the remaining 7% of SNPs (A to T and T to A) can also be corrected (orange highlight).

From	To	% of SNPs in ClinVar	Equivalent to		Base-editing route for correction
A	T	7	T	A	A to G (ABE), G to C (CGBE), C to T (CBE)
	G	48		C	ABE
	C	15		G	C to G (CGBE), G to A (CBE)
G	A	6	C	T	CBE
	T	14		A	A to G (ABE), G to C (CGBE)
	C	11		G	CGBE

Supplementary Table 2. Target protospacer sequences used in this study. Targeted C's are underlined. PAMs are in bold.

Name	Target Sequences and PAMs
EMX1	GAGTCCGAGCAGAAGAAGAA**GGG**
FANCF	GGAATCCCTTCTGCAGCACC**TGG**
HEK2-1	GAACACAAAGCATAGACTGC**GGG**
HEK2-2	GAGCACCACACCCTAAACTA**TGG**
HEK2-3	GGAAACGGATAGTTCTGAAA**GGG**
HEK2-4	CTTAACTATTTGTATTCCAC**TGG**
HEK2-5	CTTCCCAAGTGAGAAGCCAG**TGG**
HEK2-6	CCAGCCCGCTGGCCCTGTAA**AGG**
HEK2-7	CATTCCGTTATTTTACATAT**TGG**
HEK2-8	GTTTCCTTTACAGGGCCAGC**GGG**
HEK2-9	ATACGCACAGTTTGACAGAT**GGG**
HEK2-10	GCTGGCCCTGTAAAGGAAAC**TGG**
HEK2-11	GCATGCGTGTGTGTTTAAGC**TGG**
HEK2-12	TTGGGCTGCAGTAACTTGAA**GGG**
HEK2-13	TCTTTCAAGCAGGTGATTAC**AGG**
HEK2-14	AGTTTCCTTTACAGGGCCAG**CGG**
HEK2-15	GAGGTCGTGGCTGAGCACAA**GGG**
HEK2-16	GGCCTCTATTGTTGGTAGAA**TGG**
HEK3	GGCCCAGACTGAGCACGTGA**TGG**
HEK4	GGCACTGCGGCTGGAGGTGG**GGG**
RNF2-1	GTCATCTTAGTCATTACCTG**AGG**
RNF2-2	CACACACACTTAGAATCTGT**GGG**
RNF2-3	ACACACACACTTAGAATCTG**TGG**
GJB2	GGACACGAAGATCAGCTGCA**GGG**
ADRB2	CCCTTTCCTGCGTGACGTCG**TGG**
MYBPC3	CCCTTTCCTGCGTGACGTCG**TGG**
GAL 292	GAAGTCGTTGTCAAACAGGA**AGG**
VEGFA-1	GATGTCTGCAGGCCAGATGA**GGG**

Supplementary Table 3. HTS Primers used in this study. PCR1 of the iSeq sample preparation uses primers listed below. The red parts are common to all HTS primers. They serve as priming regions for PCR2 (barcoding). Depending on the barcoding primers used, the red parts should be varied accordingly. The black parts of the HTS primers are unique to the genomic locus that the primers are meant to amplify.

Name	Primer Sequence	Used for
LC041	CTTTCCCTACACGACGCTCTTCCGATCTNNNNNNATGTGG GCTGCCTAGAAAGG	Forward primer, HTS, HEK3
LC042	GGAGTTCAGACGTGTGCTCTTCCGATCTCCCAGCCAAACT TGTCAACC	Reverse primer, HTS, HEK3
LC183	CTTTCCCTACACGACGCTCTTCCGATCTNNNNNNCCAGCC CCATCTGTCAAACT	Forward primer, HTS, HEK2-1, -6, -7, -8, -10, -12, -14
LC184	GGAGTTCAGACGTGTGCTCTTCCGATCTTGAATGGATTCC TTGGAAACAATGA	Reverse primer, HTS, HEK2-1, -4, -5, -9
LC231	CTTTCCCTACACGACGCTCTTCCGATCTNNNNNNTAGTCT TTCAAGCAGGTGATTACAGG	Forward primer 2, HTS, HEK2-1, -4, -5, -9
LC232	GGAGTTCAGACGTGTGCTCTTCCGATCTCTACCAACAATA GAGGCCCATTAA	Reverse primer 2, HTS, HEK2-1, -6, -7, -8, -10, -12, -14
LC256	CTTTCCCTACACGACGCTCTTCCGATCTNNNNNNCAGCTC AGCCTGAGTGTTGA	Forward primer, HTS, EMX1
LC257	GGAGTTCAGACGTGTGCTCTTCCGATCTCTCGTGGGTTTG TGGTTGC	Reverse primer, HTS, EMX1
LC258	CTTTCCCTACACGACGCTCTTCCGATCTNNNNNNACGTAG GAATTTTGGTGGGACA	Forward primer, HTS, RNF2-1
LC259	GGAGTTCAGACGTGTGCTCTTCCGATCTACGTCTCATATG CCCCTTGG	Reverse primer, HTS, RNF2-1
LC260	CTTTCCCTACACGACGCTCTTCCGATCTNNNNNNCATTGC AGAGAGGCGTATCA	Forward primer, HTS, FANCF
LC261	GGAGTTCAGACGTGTGCTCTTCCGATCTGGGGTCCCAGGT GCTGAC	Reverse primer, HTS, FANCF
LC262	CTTTCCCTACACGACGCTCTTCCGATCTNNNNNNGAACCC AGGTAGCCAGAGAC	Forward primer, HTS, HEK4
LC263	GGAGTTCAGACGTGTGCTCTTCCGATCTTCCTTTCAACCC GAACGGAG	Reverse primer, HTS, HEK4
LC266	CTTTCCCTACACGACGCTCTTCCGATCTNNNNNNGCAGGG CTAATAAATGGTCCTGTG	Forward Primer, HTS, HEK2-15
LC267	GGAGTTCAGACGTGTGCTCTTCCGATCTCGGTCCTACATC ACCCCTTCT	Reverse Primer, HTS, HEK2-15
LC268	CTTTCCCTACACGACGCTCTTCCGATCTNNNNNNCACATT GGAGTGTCCAGTTGGT	Forward Primer, HTS, HEK2-3
LC269	GGAGTTCAGACGTGTGCTCTTCCGATCTGGAAGACAACAT GTTTCCCCCAT	Reverse Primer, HTS, HEK2-3
LC270	CTTTCCCTACACGACGCTCTTCCGATCTNNNNNNACAGGG GAAGGTAAGTTTGGG	Forward Primer, HTS, HEK2-2
LC271	GGAGTTCAGACGTGTGCTCTTCCGATCTAGGTCTCAGATA CAGCCTGA	Reverse Primer, HTS, HEK2-2
LC272	CTTTCCCTACACGACGCTCTTCCGATCTNNNNNNTCGGTG AATTTTAATTGAGTTGTGT	Forward Primer, HTS, HEK2-11
LC273	GGAGTTCAGACGTGTGCTCTTCCGATCTACTAGCTGAGCT TTTGTGTCCA	Reverse Primer, HTS, HEK2-11
LC274	CTTTCCCTACACGACGCTCTTCCGATCTNNNNNNTGCTCC CCTCAGCATTCAGC	Forward Primer, HTS, HEK2-13

LC275	GGAGTTCAGACGTGTGCTCTTCCGATCTACTGGAACACAA AGCATAGACTGC	Reverse Primer, HTS, HEK2-13
LC276	CTTTCCCTACACGACGCTCTTCCGATCTNNNNNNCATTCC GTTATTTTACATATTGGGC	Forward Primer, HTS, HEK2-16
LC277	GGAGTTCAGACGTGTGCTCTTCCGATCTACATTTGGGCTT CTTTCTAGTTGA	Reverse Primer, HTS, HEK2-16
LC302	CTTTCCCTACACGACGCTCTTCCGATCTNNNNNNAAAATC TCAGCGCTTTCGTCC	Forward Primer, HTS, RNF2-2,-3
LC303	GGAGTTCAGACGTGTGCTCTTCCGATCTGTCTTAGGAAGT TTCAGGGCTGG	Reverse Primer, HTS, RNF2-2,-3
LC402	CTTTCCCTACACGACGCTCTTCCGATCTNNNNNNCAAAGG ACCCCAGTCACTCC	Forward primer, HTS, VEGFA
LC403	GGAGTTCAGACGTGTGCTCTTCCGATCTTTTGCTCCTGGA CCCCCTAT	Reverse primer, HTS, VEGFA
HEK2_1_F	CTTTCCCTACACGACGCTCTTCCGATCTNNNNNNAGTGTG GAGAGTGAGTAAGCCAG	Forward primer HEK2_OFT1
HEK2_1_R	GGAGTTCAGACGTGTGCTCTTCCGATCTTCTAACGGTAGG ATGATTTCAGGCA	Reverse primer HEK2_OFT1
HEK2_2_F	CTTTCCCTACACGACGCTCTTCCGATCTNNNNNNAGGAAA AGCAACGTGAGCCTTAAA	Forward primer HEK2_OFT2
HEK2_2_R	GGAGTTCAGACGTGTGCTCTTCCGATCTTCCTCCTAAAAG CCTCCATTCACC	Reverse primer HEK2_OFT2
HEK3_1_F	CTTTCCCTACACGACGCTCTTCCGATCTNNNNNNTCCCCT GTTGACCTGGAGAA	Forward primer HEK3_OFT1
HEK3_1_R	GGAGTTCAGACGTGTGCTCTTCCGATCTCACTGTACTTGC CCTGACCA	Reverse primer HEK3_OFT1
HEK3_2_F	CTTTCCCTACACGACGCTCTTCCGATCTNNNNNNTTGGTG TTGACAGGGAGCAA	Forward primer HEK3_OFT2
HEK3_2_R	GGAGTTCAGACGTGTGCTCTTCCGATCTCTGAGATGTGGG CAGAAGGG	Reverse primer HEK3_OFT2
HEK3_3_F	CTTTCCCTACACGACGCTCTTCCGATCTNNNNNNTGAGAG GGAACAGAAGGGCT	Forward primer HEK3_OFT3
HEK3_3_R	GGAGTTCAGACGTGTGCTCTTCCGATCTGTCCAAAGGCCC AAGAACCT	Reverse primer HEK3_OFT3
HEK3_4_F	CTTTCCCTACACGACGCTCTTCCGATCTNNNNNNCCTAGC ACTTTGGAAGGTCG	Forward primer HEK3_OFT4
HEK3_4_R	GGAGTTCAGACGTGTGCTCTTCCGATCTGCTCATCTTAAT CTGCTCAGCC	Reverse primer HEK3_OFT4
HEK3_5_F	CTTTCCCTACACGACGCTCTTCCGATCTNNNNNNAAAGGA GCAGCTCTTCCTGG	Forward primer HEK3_OFT5
HEK3_5_R	GGAGTTCAGACGTGTGCTCTTCCGATCTGTCTGCACCATC TCCCACAA	Reverse primer HEK3_OFT5
HEK4_2_F	CTTTCCCTACACGACGCTCTTCCGATCTNNNNNNGGACCC TATTCGGGGCATGG	Forward primer HEK4_OFT2
HEK4_2_R	GGAGTTCAGACGTGTGCTCTTCCGATCTTGATAGAAGCGG ACCCCACAT	Reverse primer HEK4_OFT2
HEK4_3_F	CTTTCCCTACACGACGCTCTTCCGATCTNNNNNNGTGTCC CATGGAGGCTGCT	Forward primer HEK4_OFT3
HEK4_3_R	GGAGTTCAGACGTGTGCTCTTCCGATCTCCAGGCTGTGGT AGGGACTC	Reverse primer HEK4_OFT3
HEK4_5_F	CTTTCCCTACACGACGCTCTTCCGATCTNNNNNNCGTAGC TTCAGGACGGCCC	Forward primer HEK4_OFT5
HEK4_5_R	GGAGTTCAGACGTGTGCTCTTCCGATCTTTCCCTGGTCCA CACTGACAC	Reverse primer HEK4_OFT5
HEK4_7_F	CTTTCCCTACACGACGCTCTTCCGATCTNNNNNNGCACCT GTGAAACCACAGCC	Forward primer HEK4_OFT7
HEK4_7_R	GGAGTTCAGACGTGTGCTCTTCCGATCTCCCCTTGCACTC CCTGTCTT	Reverse primer HEK4_OFT7

HEK4_11_F	CTTTCCCTACACGACGCTCTTCCGATCTNNNNNNCCTGGAGGTCTACTGGACGGG	Forward primer HEK4_OFT11
HEK4_11_R	GGAGTTCAGACGTGTGCTCTTCCGATCTCACGGCCCCTCCCAGTTTATAG	Reverse primer HEK4_OFT11
HEK4_13_F	CTTTCCCTACACGACGCTCTTCCGATCTNNNNNNCAGAGAGGTGAGAGGCAGGC	Forward primer HEK4_OFT13
HEK4_13_R	GGAGTTCAGACGTGTGCTCTTCCGATCTCTGCTGGGCCTAAGCCATCT	Reverse primer HEK4_OFT13
HEK4_18_F	CTTTCCCTACACGACGCTCTTCCGATCTNNNNNNGGAGACACGGCTGAAGATCTGA	Forward primer HEK4_OFT18
HEK4_18_R	GGAGTTCAGACGTGTGCTCTTCCGATCTTCCCCAGGCAACCCAAAGAG	Reverse primer HEK4_OFT18
EMX1_1_F	CTTTCCCTACACGACGCTCTTCCGATCTNNNNNNTGCCCAATCATTGATGCTTTT	Forward primer EMX1_1
EMX1_1_R	GGAGTTCAGACGTGTGCTCTTCCGATCTAGAAACATTTACCATAGACTATCACCT	Reverse primer EMX1_1
EMX1_2_F	CTTTCCCTACACGACGCTCTTCCGATCTNNNNNNAGTAGCCTCTTTCTCAATGTGC	Forward primer EMX1_2
EMX1_2_R	GGAGTTCAGACGTGTGCTCTTCCGATCTGCTTTCACAAGGATGCAGTCT	Reverse primer EMX1_2
EMX1_3_F	CTTTCCCTACACGACGCTCTTCCGATCTNNNNNNGAGCTAGACTCCGAGGGGA	Forward primer EMX1_3
EMX1_3_R	GGAGTTCAGACGTGTGCTCTTCCGATCTTCCTCGTCCTGCTCTCACTT	Reverse primer EMX1_3
EMX1_4_F	CTTTCCCTACACGACGCTCTTCCGATCTNNNNNNAGAGGCTGAAGAGGAAGACCA	Forward primer EMX1_4
EMX1_4_R	GGAGTTCAGACGTGTGCTCTTCCGATCTGGCCCAGCTGTGCATTCTAT	Reverse primer EMX1_4
EMX1_6_F	CTTTCCCTACACGACGCTCTTCCGATCTNNNNNNCCAAGAGGGCCAAGTCCTG	Forward primer EMX1_6
EMX1_6_R	GGAGTTCAGACGTGTGCTCTTCCGATCTCAGCGAGGAGTGACAGCC	Reverse primer EMX1_6
EMX1_7_F	CTTTCCCTACACGACGCTCTTCCGATCTNNNNNNCACTCCACCTGATCTCGGGG	Forward primer EMX1_7
EMX1_7_R	GGAGTTCAGACGTGTGCTCTTCCGATCTCGAGGAGGGAGGGAGCAG	Reverse primer EMX1_7
EMX1_8_F	CTTTCCCTACACGACGCTCTTCCGATCTNNNNNNACCACAAATGCCCAAGAGAC	Forward primer EMX1_8
EMX1_8_R	GGAGTTCAGACGTGTGCTCTTCCGATCTGACACAGTCAAGGGCCGG	Reverse primer EMX1_8
EMX1_9_F	CTTTCCCTACACGACGCTCTTCCGATCTNNNNNNCCCACCTTTGAGGAGGCAAA	Forward primer EMX1_9
EMX1_9_R	GGAGTTCAGACGTGTGCTCTTCCGATCTTTCCATCTGAGAAGAGAGTGGT	Reverse primer EMX1_9
EMX1_10_F	CTTTCCCTACACGACGCTCTTCCGATCTNNNNNNGTCATACCTTGGCCCTTCCT	Forward primer EMX1_10
EMX1_10_R	GGAGTTCAGACGTGTGCTCTTCCGATCTTCCCTAGGCCCACACCAG	Reverse primer EMX1_10
FANCF_1_F	CTTTCCCTACACGACGCTCTTCCGATCTNNNNNNCTCCGGGGGCCATTCAGAAA	Forward primer FANCF_1
FANCF_1_R	GGAGTTCAGACGTGTGCTCTTCCGATCTCCTCGTGACCGACACACAGT	Reverse primer FANCF_1
FANCF_5_F	CTTTCCCTACACGACGCTCTTCCGATCTNNNNNNCTCCAGTACAGGGGCTTTTGC	Forward primer FANCF_5
FANCF_5_R	GGAGTTCAGACGTGTGCTCTTCCGATCTGAAGCAGGAATTTTCCCAGCACT	Reverse primer FANCF_5
FANCF_6_F	CTTTCCCTACACGACGCTCTTCCGATCTNNNNNNCGCAGCTCTCGCACACATAG	Forward primer FANCF_6
FANCF_6_R	GGAGTTCAGACGTGTGCTCTTCCGATCTGAGCCCAGTCTCGTCCGATG	Reverse primer FANCF_6

FANCF_7_F	CTTTCCCTACACGACGCTCTTCCGATCTNNNNNNTTTTCTCCACGGAGGGGGC	Forward primer FANCF_7
FANCF_7_R	GGAGTTCAGACGTGTGCTCTTCCGATCTGGGCCCGTATTGGTTAGCTC	Reverse primer FANCF_7
FANCF_9_F	CTTTCCCTACACGACGCTCTTCCGATCTNNNNNNGAGACCTAGGTGCTGCGGAA	Forward primer FANCF_9
FANCF_9_R	GGAGTTCAGACGTGTGCTCTTCCGATCTTGCAACAGGGAAGTCCACCG	Reverse primer FANCF_9
FANCF_11_F	CTTTCCCTACACGACGCTCTTCCGATCTNNNNNNAGCTCGAGGCCGAGAATTACC	Forward primer FANCF_11
FANCF_11_R	GGAGTTCAGACGTGTGCTCTTCCGATCTTGTCGTCCTTCCTTTCGGTCA	Reverse primer FANCF_11
FANCF_15_F	CTTTCCCTACACGACGCTCTTCCGATCTNNNNNNGGGGGTCTAGAGCATCGGG	Forward primer FANCF_15
FANCF_15_R	GGAGTTCAGACGTGTGCTCTTCCGATCTAGAAGTTTGGGAAGGTCCCACC	Reverse primer FANCF_15
GAL292_F	CTTTCCCTACACGACGCTCTTCCGATCTNNNNNNGTATGGGGCAGTGAGTGCTT	Forward primer, HTS PCR1,GAL292
GAL292_R	GGAGTTCAGACGTGTGCTCTTCCGATCTGAGGTCTAGCCACCCTCCTC	Reverse primer, HTS PCR1,GAL292
GJB2_F	CTTTCCCTACACGACGCTCTTCCGATCTNNNNNNCAGGCCGACTTTGTCTGCAACA	Forward primer, HTS PCR1,GJB2
GJB2_R	GGAGTTCAGACGTGTGCTCTTCCGATCTGAAGGCGGCTTCGAAGATGACC	Reverse primer, HTS PCR1,GJB2
ADRB2_F	CTTTCCCTACACGACGCTCTTCCGATCTNNNNNNCTTCTTGCTGGCACCCAAT	Forward primer, HTS PCR1,ADRB2
ADRB2_R	GGAGTTCAGACGTGTGCTCTTCCGATCTAACTTGGCAATGGCTGTGAT	Reverse primer, HTS PCR1,ADRB2
MYBPC3_F	CTTTCCCTACACGACGCTCTTCCGATCTNNNNNNCGGCCACTCCCAGTCTCCTTTA	Forward primer, HTS PCR1,MYBPC3
MYBPC3_R	GGAGTTCAGACGTGTGCTCTTCCGATCTGTCCTTGGTGGACACCTCAC	Reverse primer, HTS PCR1,MYBPC3

DNA sequences of CGBE constructs and BER proteins

>ACX, rXRCC1

rAPOBEC in red; nCas9 in green; rXRCC1 in blue; His-Tag in orange.

atgagctcagagactggcccagtggctgtggaccccacattgagacggcggatcgagccccа
tgagtttgaggtattcttcgatccgagagagctccgcaaggagacctgcctgctttacgaaa
ttaattgggggggccggcactccatttggcgacatacatcacagaacactaacaagcacgtc
gaagtcaacttcatcgagaagttcacgacagaaagatatttctgtccgaacacaaggtgcag
cattacctggtttctcagctggagcccatgcggcgaatgtagtagggccatcactgaattcc
tgtcaaggtatccccacgtcactctgtttatttacatcgcaaggctgtaccaccacgctgac
cccgcaatcgacaaggcctgcgggatttgatctcttcaggtgtgactatccaaattatgac
tgagcaggagtcaggatactgctggagaaactttgtgaattatagcccgagtaatgaagccc
actggcctaggtatccccatctgtgggtacgactgtacgttcttgaactgtactgcatcata
ctgggcctgcctccttgtctcaacattctgagaaggaagcagccacagctgacattctttac
catcgctcttcagtcttgtcattaccagcgactgcccccacacattctctgggccaccgggt
tgaaaagcggcagcgagactcccgggacctcagagtccgccacacccgaaagtgataaaaag
tattctattggtttagccatcggcactaattccgttggatgggctgtcataaccgatgaata
caaagtaccttcaaagaaatttaaggtgttggggaacacagaccgtcattcgattaaaaaga
atcttatcggtgccctcctattcgatagtggcgaaacggcagaggcgactcgcctgaaacga
accgctcggagaaggtatacacgtcgcaagaaccgaatatgttacttacaagaaatttttag
caatgagatggccaaagttgacgattctttctttcaccgtttggaagagtccttccttgtcg
aagaggacaagaaacatgaacggcaccccatctttggaaacatagtagatgaggtggcatat
catgaaaagtacccaacgatttatcacctcagaaaaaagctagttgactcaactgataaagc
ggacctgaggttaatctacttggctcttgcccatatgataaagttccgtgggcactttctca
ttgagggtgatctaaatccggacaactcggatgtcgacaaactgttcatccagttagtacaa
acctataatcagttgtttgaagagaaccctataaatgcaagtggcgtggatgcgaaggctat
tcttagcgcccgcctctctaaatcccgacggctagaaaacctgatcgcacaattacccggag
agaagaaaaatgggttgttcggtaaccttatagcgctctcactaggcctgacaccaaatttt
aagtcgaacttcgacttagctgaagatgccaaattgcagcttagtaaggacacgtacgatga
cgatctcgacaatctactggcacaaattggagatcagtatgcggacttatttttggctgcca
aaaaccttagcgatgcaatcctcctatctgacatactgagagttaatactgagattaccaag
gcgccgttatccgcttcaatgatcaaaaggtacgatgaacatcaccaagacttgacacttct
caaggccctagtccgtcagcaactgcctgagaaatataaggaaatattctttgatcagtcga
aaaacgggtacgcaggttatattgacggcggagcgagtcaagaggaattctacaagtttatc
aaacccatattagagaagatggatgggacggaagagttgcttgtaaaactcaatcgcgaaga
tctactgcgaaagcagcggactttcgacaacggtagcattccacatcaaatccacttaggcg
aattgcatgctatacttagaaggcaggaggatttttatccgttcctcaaagacaatcgtgaa
aagattgagaaaatcctaacctttcgcataccttactatgtgggacccctggcccgagggaa
ctctcggttcgcatggatgacaagaaagtccgaagaaacgattactccatggaattttgagg
aagttgtcgataaaggtgcgtcagctcaatcgttcatcgagaggatgaccaactttgacaag
aatttaccgaacgaaaaagtattgcctaagcacagtttactttacgagtatttcacagtgta
caatgaactcacgaaagttaagtatgtcactgagggcatgcgtaaacccgcctttctaagcg
gagaacagaagaaagcaatagtagatctgttattcaagaccaaccgcaaagtgacagttaag
caattgaaagaggactactttaagaaaattgaatgcttcgattctgtcgagatctccggggt
agaagatcgatttaatgcgtcacttggtacgtatcatgacctcctaaagataattaaagata
aggacttcctggataacgaagagaatgaagatatcttagaagatatagtgttgactcttacc

ctctttgaagatcgggaaatgattgaggaaagactaaaaacatacgctcacctgttcgacga
taaggttatgaaacagttaaagaggcgtcgctatacgggctggggacgattgtcgcggaaac
ttatcaacgggataagagacaagcaaagtggtaaaactattctcgattttctaaagagcgac
ggcttcgccaataggaactttatgcagctgatccatgatgactctttaaccttcaaagagga
tatacaaaaggcacaggtttccggacaaggggactcattgcacgaacatattgcgaatcttg
ctggttcgccagccatcaaaaagggcatactccagacagtcaaagtagtggatgagctagtt
aaggtcatgggacgtcacaaaccggaaaacattgtaatcgagatggcacgcgaaaatcaaac
gactcagaaggggcaaaaaaacagtcgagagcggatgaagagaatagaagagggtattaaag
aactgggcagccagatcttaaaggagcatcctgtggaaaatacccaattgcagaacgagaaa
ctttacctctattacctacaaaatggaagggacatgtatgttgatcaggaactggacataaa
ccgtttatctgattacgacgtcgatcacattgtaccccaatcctttttgaaggacgattcaa
tcgacaataaagtgcttacacgctcggataagaaccgagggaaaagtgacaatgttccaagc
gaggaagtcgtaaagaaaatgaagaactattggcggcagctcctaaatgcgaaactgataac
gcaaagaaagttcgataacttaactaaagctgagaggggtggcttgtctgaacttgacaagg
ccggatttattaaacgtcagctcgtggaaacccgccaaatcacaaagcatgttgcacagata
ctagattcccgaatgaatacgaaatacgacgagaacgataagctgattcgggaagtcaaagt
aatcactttaaagtcaaaattggtgtcggacttcagaaaggattttcaattctataaagtta
gggagataaataactaccaccatgcgcacgacgcttatcttaatgccgtcgtagggaccgca
ctcattaagaaatacccgaagctagaaagtgagtttgtgtatggtgattacaaagtttatga
cgtccgtaagatgatcgcgaaaagcgaacaggagataggcaaggctacagccaaatacttct
tttattctaacattatgaatttctttaagacggaaatcactctggcaaacggagagatacgc
aaacgacctttaattgaaaccaatggggagacaggtgaaatcgtatgggataagggccggga
cttcgcgacggtgagaaaagttttgtccatgccccaagtcaacatagtaaagaaaactgagg
tgcagaccggagggttttcaaaggaatcgattcttccaaaaaggaatagtgataagctcatc
gctcgtaaaaaggactgggacccgaaaaagtacggtggcttcgatagccctacagttgccta
ttctgtcctagtagtggcaaaagttgagaagggaaaatccaagaaactgaagtcagtcaaag
aattattggggataacgattatggagcgctcgtcttttgaaaagaaccccatcgacttcctt
gaggcgaaaggttacaaggaagtaaaaaaggatctcataattaaactaccaaagtatagtct
gtttgagttagaaaatggccgaaaacggatgttggctagcgccggagagcttcaaaagggga
acgaactcgcactaccgtctaaatacgtgaatttcctgtatttagcgtcccattacgagaag
ttgaaaggttcacctgaagataacgaacagaagcaactttttgttgagcagcacaaacatta
tctcgacgaaatcatagagcaaatttcggaattcagtaagagagtcatcctagctgatgcca
atctggacaaagtattaagcgcatacaacaagcacagggataaacccatacgtgagcaggcg
gaaaatattatccatttgtttactcttaccaacctcggcgctccagccgcattcaagtattt
tgacacaacgatagatcgcaaacgatacacttctaccaaggaggtgctagacgcgacactga
ttcaccaatccatcacgggattatatgaaactcggatagatttgtcacagcttgggggtgac
tctggtggttctcctgagataagcctccgccacgtcgtaagttgctccagtcaagactccac
tcatcgagctgagaacttgttgaaagccgacacgtacagaaagtggaggagcgccaaagcag
gggaaaaaacgatatccgtcgttttgcagctggagaaagaggaacagatacacagcgttgac
atcggaaacgatggcagtgcatttgtagaagttttggttggtagtagcgcaggaggcgctac
ggccggtgagcaagactatgaagtgctcttggtgaccagctctttcatgtccccgtctgaaa
gccgaagcgggtctaacccaaatagggtccgaatattcgggccggataaattggttagagcc
gctgctgagaagaggtgggaccgcgttaagattgtatgttcacagccgtattccaaggacag
cccgtatgggctctcatttgttaaatttcatagcccccagataaggacgaggccgaagctc
cgtcacagaaggtgactgtaactaaacttggacaattccgagtcaaagaggaggatgattca
gcgaatagtctccggcccggtgcgttgtttttcaatcgcatcaacaaagctgccagcgcaag
tgcctcagaccctgccgggcctagttatgccgcagcaactctgcaagccagttcagctgcgt

caagtgctctccccgtcccaaaggttggcggttcttcctcaaaactgcaagagcctccaaag
ggcaagcggaagcttgatctgggattggaggattcaaaacctccctctaaaccttcagcggg
acctgcagctctgaagagacctaaactcccggtcccgtctcggacgccggccgccacaccag
caagtactcctgcgcagaaggcagttcccgggaaaccaagaggtgagggcaccgaaccgcgc
ggggctcgggctggtccccaagagttggggaagattctccaaggagtggtggtcgtacttag
tggcttccagaatcctttccgctctgaacttcgcgacaaagcgttggagcttggtgccaaat
acagaccagactggacccctgactctacccatttgatctgtgcatttgccaatactccgaaa
tatagtcaagttcttgggcttggtggtagaattgttagaaaagagtgggtattggactgcta
ccgaatgcgaagaaggctgccttcacgaaggtacctcatggctggccttgggtcctcatctg
aggacgagggagatagccactctgagtccggcgaggatgaagcgcctaaattgccacggaag
aggccccaaccgaaggctaagacgcaagccgctgggccaagttctccaccaagacctccaac
gccagaagagaccaaagccccatcacccggcccacaggataactccgacactgatggggagc
aaagcgagggtcgagataatggagcggaggactccggtgatactgaagatgaactcaggcgc
gtggcgaagcaacgggaacaaaggcagccgcctgcaccagaggaaaatggagaggatccata
cgcgggtagtacagacgagaatactgattcagaagctccttccgaggccgacctccccatac
ccgagctccccgacttcttccagggcaagcatttctttctctacggcgagttccccggagac
gaacgaaggaaactcatccgatacgttacagcatttaatggcgagttggaagattacatgtc
tgatagggtacagtttgtgatcaccgcgcaagagtgggatcctaattttgaggaggccttga
tggaaaatccctctcttgcttttgttcggcccagatggatatattcttgcaatgaaaagcaa
aagttgctccctcatcagttgtatggggtcgtaccccaggcatctggtggttctcccaagaa
gaagaggaaagtctaaccggtcatcatcaccatcaccattga

>ACX, rPB(8kD)

rAPOBEC in red; nCas9 in green; rPB(8kD) in blue; His-Tag in orange.

atgagctcagagactggcccagtggctgtggaccccacattgagacggcggatcgagcccca
tgagtttgaggtattcttcgatccgagagagctccgcaaggagacctgcctgctttacgaaa
ttaattgggggggccggcactccatttggcgacatacatcacagaacactaacaagcacgtc
gaagtcaacttcatcgagaagttcacgacagaaagatatttctgtccgaacacaaggtgcag
cattacctggtttctcagctggagcccatgcggcgaatgtagtagggccatcactgaattcc
tgtcaaggtatccccacgtcactctgtttatttacatcgcaaggctgtaccaccacgctgac
ccccgcaatcgacaaggcctgcgggatttgatctcttcaggtgtgactatccaaattatgac
tgagcaggagtcaggatactgctggagaaactttgtgaattatagcccgagtaatgaagccc
actggcctaggtatccccatctgtgggtacgactgtacgttcttgaactgtactgcatcata
ctgggcctgcctccttgtctcaacattctgagaaggaagcagccacagctgacattctttac
catcgctcttcagtcttgtcattaccagcgactgcccccacacattctctgggccaccgggt
tgaaaagcggcagcgagactcccgggacctcagagtccgccacacccgaaagtgataaaaag
tattctattggtttagccatcggcactaattccgttggatgggctgtcataaccgatgaata
caaagtaccttcaaagaaatttaaggtgttggggaacacagaccgtcattcgattaaaaaga
atcttatcggtgccctcctattcgatagtggcgaaacggcagaggcgactcgcctgaaacga
accgctcggagaaggtatacacgtcgcaagaaccgaatatgttacttacaagaaattttag
caatgagatggccaaagttgacgattctttctttcaccgtttggaagagtccttccttgtcg
aagaggacaagaaacatgaacggcaccccatctttggaaacatagtagatgaggtggcatat
catgaaaagtacccaacgatttatcacctcagaaaaagctagttgactcaactgataaagc
ggacctgaggttaatctacttggctcttgcccatatgataaagttccgtgggcactttctca
ttgagggtgatctaaatccggacaactcggatgtcgacaaactgttcatccagttagtacaa
acctataatcagttgtttgaagagaaccctataaatgcaagtggcgtggatgcgaaggctat

tcttagcgcccgcctctctaaatcccgacggctagaaaacctgatcgcacaattacccggag
agaagaaaaatgggttgttcggtaaccttatagcgctctcactaggcctgacaccaaatttt
aagtcgaacttcgacttagctgaagatgccaaattgcagcttagtaaggacacgtacgatga
cgatctcgacaatctactggcacaaattggagatcagtatgcggacttattttggctgcca
aaaaccttagcgatgcaatcctcctatctgacatactgagagttaatactgagattaccaag
gcgccgttatccgcttcaatgatcaaaaggtacgatgaacatcaccaagacttgacacttct
caaggccctagtccgtcagcaactgcctgagaaatataaggaaatattctttgatcagtcga
aaaacgggtacgcaggttatattgacggcggagcgagtcaagaggaattctacaagtttatc
aaacccatattagagaagatggatgggacggaagagttgcttgtaaaactcaatcgcgaaga
tctactgcgaaagcagcggactttcgacaacggtagcattccacatcaaatccacttaggcg
aattgcatgctatacttagaaggcaggaggattttatccgttcctcaaagacaatcgtgaa
aagattgagaaaatcctaacctttcgcataccttactatgtgggacccctggcccgagggaa
ctctcggttcgcatggatgacaagaaagtccgaagaaacgattactccatggaattttgagg
aagttgtcgataaaggtgcgtcagctcaatcgttcatcgagaggatgaccaactttgacaag
aatttaccgaacgaaaaagtattgcctaagcacagtttactttacgagtatttcacagtgta
caatgaactcacgaaagttaagtatgtcactgagggcatgcgtaaacccgcctttctaagcg
gagaacagaagaaagcaatagtagatctgttattcaagaccaaccgcaaagtgacagttaag
caattgaaagaggactactttaagaaaattgaatgcttcgattctgtcgagatctccggggt
agaagatcgatttaatgcgtcacttggtacgtatcatgacctcctaaagataattaaagata
aggacttcctggataacgaagagaatgaagatatcttagaagatatagtgttgactcttacc
ctctttgaagatcgggaaatgattgaggaaagactaaaaacatacgctcacctgttcgacga
taaggttatgaaacagttaaagaggcgtcgctatacgggctggggacgattgtcgcggaaac
ttatcaacgggataagagacaagcaaagtggtaaaactattctcgattttctaaagagcgac
ggcttcgccaataggaactttatgcagctgatccatgatgactctttaaccttcaaagagga
tatacaaaaggcacaggtttccggacaaggggactcattgcacgaacatattgcgaatcttg
ctggttcgccagccatcaaaaagggcatactccagacagtcaaagtagtggatgagctagtt
aaggtcatgggacgtcacaaaccggaaaacattgtaatcgagatggcacgcgaaaatcaaac
gactcagaagggggcaaaaaaacagtcgagagcggatgaagagaatagaagagggtattaaag
aactgggcagccagatcttaaaggagcatcctgtggaaaatacccaattgcagaacgagaaa
ctttacctctattacctacaaaatggaagggacatgtatgttgatcaggaactggacataaa
ccgtttatctgattacgacgtcgatcacattgtaccccaatcctttttgaaggacgattcaa
tcgacaataaagtgcttacacgctcggataagaaccgagggaaaagtgacaatgttccaagc
gaggaagtcgtaaagaaaatgaagaactattggcggcagctcctaaatgcgaaactgataac
gcaaagaaagttcgataacttaactaaagctgagaggggtggcttgtctgaacttgacaagg
ccggatttattaaacgtcagctcgtggaaacccgccaaatcacaaagcatgttgcacagata
ctagattcccgaatgaatacgaaatacgacgagaacgataagctgattcgggaagtcaaagt
aatcactttaaagtcaaaattggtgtcggacttcagaaaggattttcaattctataaagtta
gggagataaataactaccaccatgcgcacgacgcttatcttaatgccgtcgtagggaccgca
ctcattaagaaatacccgaagctagaaagtgagtttgtgtatggtgattacaaagtttatga
cgtccgtaagatgatcgcgaaaagcgaacaggagataggcaaggctacagccaaatacttct
tttattctaacattatgaatttctttaagacggaaatcactctggcaaacggagagatacgc
aaacgacctttaattgaaaccaatggggagacaggtgaaatcgtatgggataagggccggga
cttcgcgacggtgagaaaagttttgtccatgccccaagtcaacatagtaaagaaaactgagg
tgcagaccggagggttttcaaaggaatcgattcttccaaaaaggaatagtgataagctcatc
gctcgtaaaaaggactgggacccgaaaaagtacggtggcttcgatagccctacagttgccta
ttctgtcctagtagtggcaaaagttgagaagggaaaatccaagaaactgaagtcagtcaaag
aattattggggataacgattatggagcgctcgtcttttgaaaagaaccccatcgacttcctt

gaggcgaaaggttacaaggaagtaaaaaaggatctcataattaaactaccaaagtatagtct
gtttgagttagaaaatggccgaaaacggatgttggctagcgccggagagcttcaaaaggggga
acgaactcgcactaccgtctaaatacgtgaatttcctgtatttagcgtcccattacgagaag
ttgaaaggttcacctgaagataacgaacagaagcaacttttgttgagcagcacaaacatta
tctcgacgaaatcatagagcaaatttcggaattcagtaagagagtcatcctagctgatgcca
atctggacaaagtattaagcgcatacaacaagcacagggataaacccatacgtgagcaggcg
gaaaatattatccatttgtttactcttaccaacctcggcgctccagccgcattcaagtattt
tgacacaacgatagatcgcaaacgatacacttctaccaaggaggtgctagacgcgacactga
ttcaccaatccatcacgggattatatgaaactcggatagatttgtcacagcttggggggtgac
tctggtggttctagcaaacgcaaggccccgcaggagaccctcaacggcggcatcacggacat
gctcgtggaactcgcaaactttgagaagaacgtgagccaggcgatccacaagtacaatgcat
acagaaaagcagcatctgtgatagcaaagtacccacacaaaatcaagagtggagcagaagct
aagaaattgccaggagtaggaacaaaaattgctgaaaagattgatgaattttttagcaactgg
aaaattgcgtaaactggaaaagtctggtggttctcccaagaagaagaggaaagtctaaccgg
tcatcatcaccatcaccattga

>hPB(8kD)

agcaaacggaaggcgccgcaggagactctcaacggggggaatcaccgacatgctcacagaact
cgcaaactttgagaagaacgtgagccaagctatccacaagtacaatgcttacagaaaagcag
catctgttatagcaaaatacccacacaaaataaagagtggagctgaagctaagaaattgcct
ggagtaggaacaaaaattgctgaaaagattgatgagtttttagcaactggaaaattacgtaa
actggaaaag

>hPB(14kD)

agcaaacggaaggcgccgcaggagactctcaacggggggaatcaccgacatgctcacagaact
cgcaaactttgagaagaacgtgagccaagctatccacaagtacaatgcttacagaaaagcag
catctgttatagcaaaatacccacacaaaataaagagtggagctgaagctaagaaattgcct
ggagtaggaacaaaaattgctgaaaagattgatgagtttttagcaactggaaaattacgtaa
actggaaaagattcggcaggatgatacgagttcatccatcaatttcctgactcgagttagtg
gcattggtccatctgctgcaaggaagtttgtagatgaaggaattaaaacactagaagatctc
agaaaaaatgaagataaattgaaccatcatcagcgaattgggctgaaa

>rPB(14kD)

agcaaacgcaaggccccgcaggagaccctcaacggcggcatcacggacatgctcgtggaact
cgcaaactttgagaagaacgtgagccaggcgatccacaagtacaatgcatacagaaaagcag
catctgtgatagcaaagtacccacacaaaatcaagagtggagcagaagctaagaaattgcca
ggagtaggaacaaaaattgctgaaaagattgatgaattttttagcaactggaaaattgcgtaa
actggaaaagattcgtcaggatgatacaagttcatccatcaacttcctgactcgagttactg
gcatcgggccatctgctgcaaggaagcttgtagatgaaggaattaaaacattagaagatctc
aggaaaaatgaagataaattgaaccaccatcagcgaattgggctgaaa

>hLIG3

tctaaggcagcaggtacaccaaagaagaaagctgttgtccaggctaagttgacaaccactgg
ccaggtgacttctccagtgaaaggcgcctcatttgtcaccagtaccaatccccggaaatttt
ctggcttttcagccaagcccaacaactctggggaagccccctcgagccccacccctaagaga
agtctgtcttcaagcaaatgtgaccccaggcataaggactgtctgctacgggagtttcgaaa
gttatgcgccatggtggccgataatcctagctacaacacgaagacccagatcatccaggact
tccttcggaaaggctcagcaggagatggtttccacggtgatgtgtacctaacagtgaagctg
ctgctgccaggagtcattaagactgtttacaacttgaacgataagcagattgtgaagctttt
cagtcgcattttaactgcaacccagatgatatggcacgggacctagagcagggtgacgtgt
cagagacaatcagagtcttctttgagcagagcaagtctttcccccagctgccaagagcctc
cttaccatccaggaagtggatgagttccttctgcggctgtccaagctcaccaaggaggatga
gcagcaacaggccctacaggacattgcctccaggtgtacagccaatgaccttaaatgcatca
tcaggttgatcaaacatgatctgaagatgaactcaggtgcaaaacatgtgttagacgccctt
gaccccaatgcctatgaagccttcaaagcctcgcgcaacctgcaggatgtggtggagcgggt
ccttcacaacgcgcaggaggtggagaaAgagccgggccagagacgagctctgagcgtccagg
cctcgctgatgacacctgtgcagcccatgttggcggaggcctgcaagtccgttgagtatgca
atgaagaaatgtcccaatggcatgttctctgagatcaagtacgatggagagcgagtccaggt
gcataagaatggagaccacttcagctacttcagccgcagtctcaagcccgtccttcctcaca
aggtggcccactttaaggactacattccccaggcttttcctggggccacagcatgatcttg
gattctgaagtgcttctgattgacaacaagacaggcaaaccactgccctttgggactctggg
agtacacaagaaagcagccttccaggatgctaatgtctgcctgtttgtttttgattgtatct
actttaatgatgtcagcttgatggacagacctctgtgtgagcggcggaagtttcttcatgac
aacatggttgaaattccaaaccggatcatgttctcagaaatgaagcgagtcacaaaagcttt
ggacttggctgacatgataacccgggtgatccaggagggattggagggggctggtgctgaagg
atgtgaagggtacatatgagcctgggaagcggcactggctgaaagtgaagaaagactatttg
aacgagggggccatggccgacacagctgacctggtggtccttggagccttctatgggcaagg
gagcaaaggcggcatgatgtcaatcttcctcatgggctgctacgaccctggcagccagaagt
ggtgcacagtcaccaagtgtgcaggaggccatgatgatgccacgcttgcccgcctgcagaat
gaactagacatggtgaagatcagcaaggaccccagcaaaatacccagctggttgaaggtcaa
caagatctactatcctgacttcatcgtcccagacccaaagaaagctgccgtgtgggagatca
caggggctgaattctccaaatcggaggctcatacagctgacgggatctccatccgattccct
cgctgcacccgaatccgagatgataaggactggaaatctgccactaaccttccccaactcaa
ggaactgtaccagttgtccaaggagaaggcagacttcactgtagtggctggagatgagggga
gctccactacaggggggtagcagtgaagagaataagggtccctcagggtctgctgtgtcccgc
aaggcccccagcaagccctcagccagtaccaagaaagcagaagggaagctgagtaactccaa
cagcaaagatggcaacatgcagactgcaaagccttccgctatgaaggtgggggagaagctgg
ccacaaagtcttctccagtgaaagtaggggagaagcggaaagctgctgatgagacgctgtgc
caaacaaaggtattgctggacatcttcactggggtgcggctttacttgccaccctccacacc
agacttcagccgtctcagacgctactttgtggcattcgacggggacctggtacaggaatttg
atatgacttcagccacgcacgtgctgggtagcagggacaagaaccctgcggcccagcaggtc
tccccagagtggatttgggcatgtatccggaaacggagactggtagctccctgc

>rLIG3

actttggcattcaaaacactgttcccgcggaacctttgtgcattgggtcgaaaagaattgtg
tctgtttagcgaacaacaccactggcctgcgatcagacaattttcccagtggagtgagacta

atcttctttgtggctgctgtttgttgcagcgacgaaaaccggtcctgtcttttcagcgaggt
catttgcggcctcgcgctacgcacctcatatcatggagtggttcccatgtgggactgtgcac
gggcccttgcgagatggcagagcagcgattttgtgtggattacgctaagcgcgggaccgccg
gctgcaagaagtgcaaagaaaagattgtcaagggtgtttgtcggatcgggaaggtcgtaccg
aacccttttcagaaagcgggggcgacatgaaagaatggtatcatataaaatgtatgtttga
gaaactcgaaagagctcgagcgaccacgaaaaagatcgaggatttgacagagctcgaaggct
gggaggagctggaagacaacgaaaaggaacagattagccaacacatagctgatctcagcagt
aagacagcggcaactcccaaaaagaaagctactgtgcaggccaagctcacaaccacggggca
ggtaacctctccagtgaaaggagcgagttttataacatccacgaaccctcggaagttctcag
gattttccgcagcaaaaccaaacaatagtgaacaggatccatccagccctgccccctaaaacc
tctttgtcagcatctaagtgcgaccccaaacataaagattgtctgcttcgagaatttcgaaa
gctgtgcgcgatggtagcagaaaatccgtcttataatacaaaaacgcaaataatccacgatt
ttctccagaagggttctacgggcgacggattccgcggggatgtgtatcttaccgttaaactc
ttgctgccaggggtaattaaatccgtgtacaatctgaacgataaacaaatagttaagttgtt
ttcacggatcttcaactgtaatcccgatgatatggcgagagacctggaacagggtgacgtga
gtgagacaatccgggtgtttttcgaacaatctaagagttttccgccggccgcaaaaagtctg
ctgacaattcaggaggtcgatgcattcctccttcacttgagtaaactgaccaaagaggatga
gcaacaacaggcccttcaggacattgctagtcggtgcacggcgaatgatcttaagtgtatca
taaggctcataaaacacgatctgaaaatgaattctggcgcgaagcacgttctggacgcgttg
gaccccaatgcttacgaagccttcaaggcctccagaaatttgcaggacgtcgtcgaacgggt
gttgcacaatgaacaagaggtcgagaaggatccgggccggcggagagcgctctcagttcaag
cgtctctgatgacacctgtgcagccgatgcttgcggaggcttgtaagtcaattgagtatgca
atgaaaaagtgccccaacggcatgtttagtgaaatcaaatacgacggtgagcgcgttcaggt
gcacaagaaaggcgatcacttcagctacttttcccgaagcctgaaaccggtgctgccccaca
aggtcgcccactttaaagactacatcccaaaagcatttccaggggggtcagtccatgatcctg
gactctgaggtcctgctcatcgataacaatactgggaaaccccttccgtttggaacgctggg
ggtgcacaagaaagctgcatttcaagacgcgaatgtatgtctgtttgtgtttgattgcatct
attttaatgacgtgtcattgatggacaggccgttgtgcgagagaagaaaatttcttcatgac
aatatggtagaaattcgaaacaggattatgttttcagagatgaagcaagtaactaaagccag
cgatctggccgatatgattaatcgcgtcatacgcgaggggcttgaggggctcgtcttgaagg
acgtcaagggcacgtatgaaccgggaaaaagacactggctcaaggtaaaaaaagattacctg
aatgagggagccatggctgataccgccgatctggtagttcttggagcgttctacggtcaagg
ctccaagggtgggatgatgtcaatctttctcatgggatgctatgacccggattcccaaaaat
ggtgcacagtgacaaagtgcgcgggtggtcatgatgatgcgacccttgctcggcttcaaaag
gaactcgacatggttaagatcagcaaggacccgtccaaaattcctagttggctcaagataaa
taagatatattacccagacttcatcgtcccagacccaaaaaaagcagctgtttgggaaatta
cgggtgcagagttcagtaggagtgaggcacacactgccgacgggatttcaatccgatttcca
cgatgtactaggatacgagatgataaagactggaagtctgccactaacttgccacaactgaa
agagctttaccaattgtcaaaggataaagctgatttcgcggtagtcgccggggatgaggga
gctccacaactggaggatccaatggagagaatgaggggaccgcggggtccacagtgccccgg
aaaggtccgaaaggcccaccctccaagagtagtgcatctgctaaaaagactgaacagaagct
gaatgatccatcatcacgcggtggggaaaagttggcggtaaagtcctctccggtgaaagtgg
gtatgaagcgcaaggccgcagatgagactccgggtcttacaaagaggagaagagcaagtagg
caaagagggaggcgagcaatgcggacggggcggcgg

>hXRCC1

ggaccggagatccgcctccgccatgtcgtgtcctgcagcagccaggactcgactcactgtgc
agaaaatcttctcaaggcagacacttaccgaaaatggcgggcagccaaggcaggcgagaaga
ccatctctgtggtcctacagttggagaaggaggagcagatacacagtgtggacattgggaat
gatggctcagctttcgtggaggtgctggtgggcagttcagctggaggcgctggggagcaaga
ctatgaggtccttctggtcacctcatctttcatgtccccttccgagagccgcagtggctcaa
accccaaccgcgttcgcatgtttgggcctgacaagctggtccgggcagccgccgagaagcgc
tgggaccgggtcaaaattgtttgcagccagccctacagcaaggactcccctttggcttgag
ttttgtacggtttcatagcccccagacaaagatgaggcagaggccccgtcccagaaggtga
cagtgaccaagcttggccagttccgtgtgaaggaggaggatgagagcgccaactctctgagg
ccgggggctctcttcttcagccggatcaacaagacatccccagtcacagccagcgacccggc
aggacctagctatgcagctgctaccctccaggcttctagtgctgcctcctcagcctctccag
tctccagggccataggcagcacctccaagccccaggagtctcccaaagggaagaggaagttg
gatttgaaccaagaagaaaagaagacccccagcaaaccaccagcccagctgtcgccatctgt
tcccaagagacctaaattgccagctccaactcgtaccccagccacagccccagtccctgccc
gagcacagggggcagtgacaggcaaaccccgaggagaaggcaccgagcccagacgaccccga
gctggcccagaggagctggggaagatccttcagggtgtggtagtggtgctgagtggcttcca
gaaccccttccgctccgagctgcgagataaggccctagagcttggggccaagtatcggccag
actggacccgggacagcacgcacctcatctgtgcctttgccaacacccccaagtacagccag
gtcctaggcctgggaggccgcatcgtgcgtaaggagtgggtgctggactgtcaccgcatgcg
tcggcggctgccctcccAgaggtacctcatggcagggccaggttccagcagtgaggaggatg
aggcctctcacagcggtggcagcggagatgaagcccccaagcttcctcagaagcaaccccag
accaaaaccaagcccactcaggcagctggacccagctcaccccagaagccccaacccctga
agagaccaaagcagcctcaccagtgctccaggaagatatagacattgagggggtacagtcag
aaggacaggacaatggggcggaagattctggggacacagaggatgagctgaggagggtggca
gagcagaaggaacacagactgccccctggccaggaggagaatggggaagacccgtatgcagg
ctccacggatgagaacacggacagtgaggaacaccaggagcctcctgatctgccagtccctg
agctcccagatttcttccagggcaagcacttctttctttacggggagttccctggggacgag
cggcggaaactcatccgatacgtcacagccttcaatggggagctcgaggactatatgagtga
ccgggttcagtttgtgatcacagcacaggaatgggatcccagctttgaggaggccctgatgg
acaacccctccctggcattcgttcgtccccgatggatctacagttgcaatgagaagcagaag
ttacttcctcaccagctctatggggtggtgccgcaagcc

DNA sequences of pegRNA and sgRNA used for prime editing

pegRNA (HEK2) – spacer is in blue

ccagcccgctggccctgtaagttttagagctagaaatagcaagttaaaataaggctagtccg
ttatcaacttgaaaaagtggcaccgagtcggtgctctatgctttctgttccagtttcctttta
cagggccagcggg

sgRNA (HEK2) – spacer is in blue

gcacttgtttgcagctattcgttttagagctagaaatagcaagttaaaataaggctagtccg
ttatcaacttgaaaaagtggcaccgagtcggtgc

pegRNA (HEK4) – spacer is in blue

gcggcgccccggtggcactgGttttagagctagaaatagcaagttaaaataaggctagtccg
ttatcaacttgaaaaagtggcaccgagtcggtgctccagccgcactgccaccggggcg

sgRNA (HEK4) – spacer is in blue

ccctgcctgtcatcctgcttgttttagagctagaaatagcaagttaaaataaggctagtccg
ttatcaacttgaaaaagtggcaccgagtcggtgc

pegRNA (RNF2) – spacer is in blue

tgagttacaacgaacacctcgttttagagctagaaatagcaagttaaaataaggctagtccg
ttatcaacttgaaaaagtggcaccgagtcggtgctggcagtcatcttagtcattacctgagg
tgttcgttgtaa

pegRNA (RNF2) – spacer is in blue

agttcccatgttttgcttaagttttagagctagaaatagcaagttaaaataaggctagtccg
ttatcaacttgaaaaagtggcaccgagtcggtgc

Supplementary References:

1. Doman, J. L., Raguram, A., Newby, G. A., Liu, D. R. Evaluation and minimization of Cas9-independent off-target DNA editing by cytosine base editors. Nat. Biotechnol. 38, 620-628 (2020).
2. Kim, D. et al. Genome-wide target specificities of CRISPR RNA-guided programmable deaminases. *Nat. Biotechnol.* **35**, 475-480 (2017).
3. Zeng, J. et al. Therapeutic base editing of human hematopoietic stem cells. Nat. Med. 26, 535-541 (2020).
4. Liu, D. R., Koblan, L. W. Cytosine to Guanine Base Editor. World Intellectual Property Organization (2018).
5. Kurt, I. C. et al. CRISPR C-to-G base editors for inducing targeted DNA transversions in human cells. Nat. Biotechnol. (2020). https://doi.org/10.1038/s41587-020-0609-x

6. Zhao, D. et al. New base editors change C to A in bacteria and C to G in mammalian cells. Nat. Biotechnol. (2020). https://doi.org/10.1038/s41587-020-0592-2
