# Supplementary material for: Programmable C:G to G:C genome editing with CRISPR-Cas9-directed base excision repair proteins
Source: Nat Commun. 2021 Mar 2;12:1384. doi: 10.1038/s41467-021-21559-9 (PMC7925527; doi:10.1038/s41467-021-21559-9)
Supplement: Supplementary file 3 — Reporting Summary [file 41467_2021_21559_MOESM3_ESM.pdf]

nature research

Corresponding author(s): Wei Leong Chew

Last updated by author(s): Jan 18, 2021

Reporting Summary

Nature Research wishes to improve the reproducibility of the work that we publish. This form provides structure for consistency and transparency in reporting. For further information on Nature Research policies, see Authors & Referees and the Editorial Policy Checklist .

Statistics

For all statistical analyses, confirm that the following items are present in the figure legend, table legend, main text, or Methods section.

n/a	Confirmed	
☐	☒	The exact sample size (n) for each experimental group/condition, given as a discrete number and unit of measurement
☐	☒	A statement on whether measurements were taken from distinct samples or whether the same sample was measured repeatedly
☐	☒	The statistical test(s) used AND whether they are one- or two-sided<br>*Only common tests should be described solely by name; describe more complex techniques in the Methods section.*
☒	☐	A description of all covariates tested
☒	☐	A description of any assumptions or corrections, such as tests of normality and adjustment for multiple comparisons
☐	☒	A full description of the statistical parameters including central tendency (e.g. means) or other basic estimates (e.g. regression coefficient) AND variation (e.g. standard deviation) or associated estimates of uncertainty (e.g. confidence intervals)
☐	☒	For null hypothesis testing, the test statistic (e.g. F, t, r) with confidence intervals, effect sizes, degrees of freedom and P value noted<br>*Give P values as exact values whenever suitable.*
☒	☐	For Bayesian analysis, information on the choice of priors and Markov chain Monte Carlo settings
☒	☐	For hierarchical and complex designs, identification of the appropriate level for tests and full reporting of outcomes
☒	☐	Estimates of effect sizes (e.g. Cohen's d, Pearson's r), indicating how they were calculated

Our web collection on statistics for biologists contains articles on many of the points above.

Software and code

Policy information about availability of computer code

Data collection	Sequencing data were collected via Illumina iSeq100 and manufacturer's software.
Data analysis	FASTQ files were analyzed using CRISPResso2. Statistical analyses were performed on Matlab R2016a, Microsoft Excel 2016, and Graphpad Prism 9. Weblogos were created using Weblogo 3.

For manuscripts utilizing custom algorithms or software that are central to the research but not yet described in published literature, software must be made available to editors/reviewers. We strongly encourage code deposition in a community repository (e.g. GitHub). See the Nature Research guidelines for submitting code & software for further information.

Data

Policy information about availability of data

All manuscripts must include a data availability statement. This statement should provide the following information, where applicable:

- Accession codes, unique identifiers, or web links for publicly available datasets
- A list of figures that have associated raw data
- A description of any restrictions on data availability

Sequences of ACX, rXRCC1, ACX, rPB(8kD), other BER proteins, and pegRNAs are available in the Supplementary Information. High-throughput sequencing data can be accessed via NCBI Sequence Read Archive database with SRA accession code PRJNA692655 (https://www.ncbi.nlm.nih.gov/sra/PRJNA692655) and BioProject accession code PRJNA692655 (http://www.ncbi.nlm.nih.gov/bioproject/692655). Plasmids encoding ACX, rPB(8kD) (Addgene plasmid # 165445) and ACX, rXRCC1 (Addgene plasmid # 165444) are available on Addgene.

Field-specific reporting

Please select the one below that is the best fit for your research. If you are not sure, read the appropriate sections before making your selection.

☒ Life sciences ☐ Behavioural & social sciences ☐ Ecological, evolutionary & environmental sciences

For a reference copy of the document with all sections, see nature.com/documents/nr-reporting-summary-flat.pdf

Life sciences study design

All studies must disclose on these points even when the disclosure is negative.

Sample size	A standard n ≥2 biological replicates is chosen for each gRNA:target locus experiment. These sample sizes were selected based on literature that used similar sample sizes to obtain consistently reproducible gene editing results.
Data exclusions	No data was excluded.
Replication	All data of biological replicates are included. All attempts at replication was successful. Two different researchers conducted the key biological replicates to ensure replication.<br>Consistent with other work in CRISPR editing, cells with different passage numbers were used for independent replicates.
Randomization	Experiments were conducted in identical conditions except for the tested variables. Tested variables were randomly assigned to the cell culture wells.
Blinding	Experiments were conducted in identical conditions except for the tested variables. The investigators were not blinded as consistent with past literature involving gene editing technology development via molecular and cell biology, and due to the quantitative and non-subjective nature of the data and analyses.

Reporting for specific materials, systems and methods

We require information from authors about some types of materials, experimental systems and methods used in many studies. Here, indicate whether each material, system or method listed is relevant to your study. If you are not sure if a list item applies to your research, read the appropriate section before selecting a response.

Materials & experimental systems

n/a	Involved in the study	
☒	☐	Antibodies
☐	☒	Eukaryotic cell lines
☒	☐	Palaeontology
☒	☐	Animals and other organisms
☒	☐	Human research participants
☒	☐	Clinical data

Methods

n/a	Involved in the study	
☒	☐	ChIP-seq
☒	☐	Flow cytometry
☒	☐	MRI-based neuroimaging

Eukaryotic cell lines

Policy information about cell lines

Cell line source(s)	HEK293AAV (Agilent, 240073), HTB9 cells (ATCC, 5637), eHAP cells (Horizon Discovery, C669), H9 stem cells (WiCell, WA09)
Authentication	Cells were authenticated by supplier and not further authenticated after receipt.
Mycoplasma contamination	Cells were verified to be mycoplasma negative by supplier and further validated by mycoplasma qPCR by the authors.
Commonly misidentified lines (See ICLAC register)	No commonly misidentified cell lines were used in the study
